# Poisson hurdle model-based method for clustering microbiome features

**DOI:** 10.1093/bioinformatics/btac782

**Published:** 2022-12-05

**Authors:** Zhili Qiao, Elle Barnes, Susannah Tringe, Daniel P Schachtman, Peng Liu

**Affiliations:** Department of Statistics, Iowa State University, Ames, IA 50011, USA; Department of Energy, Joint Genome Institute, Berkeley, CA 94720, USA; Department of Energy, Joint Genome Institute, Berkeley, CA 94720, USA; Environmental Genomics and Systems Biology Division, Lawrence Berkeley National Laboratory, Berkeley, CA 94720, USA; Department of Agronomy and Horticulture, University of Nebraska, Lincoln, NE 68583, USA; Department of Statistics, Iowa State University, Ames, IA 50011, USA

## Abstract

**Motivation:**

High-throughput sequencing technologies have greatly facilitated microbiome research and have generated a large volume of microbiome data with the potential to answer key questions regarding microbiome assembly, structure and function. Cluster analysis aims to group features that behave similarly across treatments, and such grouping helps to highlight the functional relationships among features and may provide biological insights into microbiome networks. However, clustering microbiome data are challenging due to the sparsity and high dimensionality.

**Results:**

We propose a model-based clustering method based on Poisson hurdle models for sparse microbiome count data. We describe an expectation–maximization algorithm and a modified version using simulated annealing to conduct the cluster analysis. Moreover, we provide algorithms for initialization and choosing the number of clusters. Simulation results demonstrate that our proposed methods provide better clustering results than alternative methods under a variety of settings. We also apply the proposed method to a sorghum rhizosphere microbiome dataset that results in interesting biological findings.

**Availability and implementation:**

R package is freely available for download at https://cran.r-project.org/package=PHclust.

**Supplementary information:**

Supplementary data are available at *Bioinformatics* online.

## 1 Introduction

Over the past two decades, the number of microbiome studies has grown rapidly due to the advancement of next-generation sequencing (NGS) technologies. With lower cost and increasing computational power, we are able to obtain tremendous amounts of data regarding the diversity and function of the microbiome from a host or a habitat. One of the most popular NGS approaches is the amplicon-based sequencing (Poretsky *et al.*, 2014) which generates data matrices of amplicon sequence variants (ASVs) or operational taxonomic units (OTUs), where ASVs and OTUs are unique taxonomic features. The ASV/OTU/taxa table is the starting point for most statistical analysis. However, these datasets present some challenges: they are high-dimensional and sparse (i.e. contain many zeroes), and there is high variability in sequencing depth across different samples (Cullen *et al.*, 2020). These data characteristics make many classic and popular data analysis approaches not directly applicable to microbiome data and call for the development of new statistical methods.

Cluster analysis has been a popular method of multivariate data analysis that helps identify relationships among high-dimensional variables. It has been widely applied to high-dimensional gene expression data (Yeung *et al.*, 2001). Applied to microbiome data, cluster analysis can help identify potential microbiome sub-communities, which give insights into how features (ASV/OTU/taxa) with similar abundance levels are grouped together. With cluster analysis, researchers can more easily identify potential species patterns from highly diverse datasets. For example, clusters may represent taxa (or strains) that are functionally related to each other (i.e. guilds) or that share sensitivity to certain environmental conditions (i.e. niche selection), which can be further probed by downstream metagenomic techniques. When applied to time series data, clustering approaches could also help identify changes in microbial community states, which could provide important insights into microbiome assembly and manipulation.

Despite the increasing demand for methods for microbiome data analysis including cluster analysis, there are not many clustering algorithms developed specifically for microbiome data. To cluster microbial features, Gloor *et al.* (2016) applied the K-means clustering, Badri *et al.* (2020) used spectral clustering, while Casero *et al.* (2017) applied the model-based Poisson clustering developed by Si *et al.* (2014) for RNA-sequencing data. To cluster samples or microbial communities, Zhang *et al.* (2017) and Lonèar-Turukalo *et al.* (2019) applied spectral clustering while the latter paper proposed an implementation of kernel principal component analysis (PCA) for data reduction. None of the above-mentioned methods take into account the excessive zeros (sparsity) in microbiome data, which is a common issue especially after rarefaction (Gloor *et al.*, 2017; McMurdie and Holmes, 2014). Such sparsity has made more and more researchers believe that the excessive zero counts in microbiome data need to be treated differently (Xu *et al.*, 2015).

In this article, we propose a model-based algorithm for clustering microbiome features based on Poisson hurdle models. The hurdle models, introduced by Cragg (1971), separately model the zero part and the non-zero part of a random variable and hence naturally allow zero inflation that often occur in microbiome count data. Hurdle model also automatically deals with the issue of dropout events. Based on mixtures of Poisson hurdle models, we developed clustering methods to group microbial features sharing similar patterns of change across different treatments/conditions.

Section 2 presents our method. We describe Poisson hurdle models for microbiome count data in Section 2.1 and propose our clustering algorithm based on mixture of Poisson hurdle models, including the expectation–maximization (EM) algorithm in Section 2.2, a stochastic modified EM algorithm in Section 2.3, an initialization method based on Kendall’s *τ* correlation in Section 2.4 and a hierarchical merging algorithm for determining number of clusters in Section 2.5. In Section 3, we compare the performance of our algorithms and other methods under a variety of simulation settings. In Section 4, we apply our proposed method to a sorghum microbiome dataset. We conclude this paper with some discussion in Section 5.

## 2 Poisson hurdle model-based clustering

Model-based clustering methods assume that data are generated by a mixture of probability distributions where each component corresponds to one cluster. Compared to traditional clustering methods such as K-means or hierarchical clustering, model-based clustering automatically offers a quantitative measure of the uncertainty of the clustering results, i.e. the probability of each feature belonging to each cluster. Extensive research has been done in model-based clustering with multivariate normal mixture distributions, see Fraley and Raftery (2002) for an excellent review. However, the count data with excessive zeros cannot be modeled directly using normal distributions. To handle the zero-inflated microbiome data, we propose a model-based clustering algorithm based on Poisson hurdle distribution.

### 2.1 Poisson hurdle distribution

Two types of statistical models have been commonly applied to modeling count data with extra zeros: zero-inflated models and hurdle models (also known as two-part models) (Hilbe, 2011). In fact, zero-inflated models are special cases of hurdle models: hurdle models can handle both zero inflation and zero deflation. Although many features of the microbiome have a lot of zeros, there are also features that are not zero-inflated and should not be modeled by zero-inflated distributions. In addition, estimates based on hurdle models tend to be more computationally stable, especially for data with small amounts of zeros (Xu *et al.*, 2015). Hence, we propose to use Poisson hurdle models for microbiome count data.

Suppose, we have a microbiome dataset with *G* features and *I* treatment groups. Let $N_{\mathrm{gij}},g=1,\ldots ,G,i=1,\ldots ,I,j=1,\ldots ,n_{i}$ denote the count data for feature *g* in replicate *j* of treatment *i*. The Poisson hurdle distribution models data by two parts separately: the zero part and the zero-truncated Poisson part. If *N_gij_* follows a Poisson hurdle distribution corresponding to a cluster *k*, then its probability mass function (pmf) is:

(1) $$f(N_{\mathrm{gij}})=\{\begin{array}{c} 1-q_{\mathrm{kij}},N_{\mathrm{gij}}=0\\ \frac{q_{\mathrm{kij}}}{1-\text{exp}(-\lambda_{\mathrm{kgij}})}\frac{\lambda_{\mathrm{kgij}}^{N_{\mathrm{gij}}}\;\text{exp}(-\lambda_{\mathrm{kgij}})}{N_{\mathrm{gij}}!},N_{\mathrm{gij}}>0 \end{array},$$

(2) $$\text{log}(\lambda_{\mathrm{kgij}})=s_{ij}+\alpha_{gk}+\mu_{ki},$$

(3) $$q_{\mathrm{kij}}=\frac{1}{1+\text{exp}[-(\gamma_{0ki}+\gamma_{1ki}s_{ij})]},\gamma_{1ki}>0,$$

where *q_kij_* is the probability of *N_gij_* in cluster *k* being positive (non-zero), and *λ_kgij_* is the mean of the Poisson distribution before zero-truncation.

In expression (2) of the Poisson mean, *s_ij_* is a normalization factor that adjusts for technical variations in sequencing depth across samples. In this article, we use the log upper-quartile estimator. This normalization method, originally proposed for RNA-seq analysis (Bullard *et al.*, 2010), has been shown to work well in microbiome datasets (Weiss *et al.*, 2017). Once estimated, *s_ij_* is treated as known. The parameter *α_gk_* represents the geometric mean abundance level in the Poisson part across all treatments for feature *g* in cluster *k*, and *μ_ki_* (with $\sum_{i=1}^{I}\mu_{ki}=0$) represents the *i*th treatment effect in abundance level for features in cluster *k*.

In expression (3), we model *q_kij_* as a logistic function of the normalization factor *s_ij_* and allow different intercepts and slopes $\gamma_{0ki},\gamma_{1ki}$ for different combinations of cluster and treatment (*k*, *i*). We further constraint $\gamma_{1ki}\geq 0$ because samples with larger sequencing depths tend to have larger non-zero proportions.

Note that in model (1), features in the same cluster have the same treatment effects (*μ_ki_*) but we allow different geometric means (*α_gk_*) across features in the same cluster. The reason is to cluster treatment effects, i.e. changes in abundance levels across treatments. Alternatively, we can also cluster features according to their abundance levels by assuming a reduced model with both the same geometric mean *α_k_* and the same treatment effects (*μ_ki_*) for all features in the same cluster. We present more details about this reduced model in Supplementary Section S1 and also have functions to implement it in our R package. The remaining part of the main text deals with the full model with *α_gk_*.

Assuming a total of *K* clusters, we model each cluster by Poisson hurdle models with cluster-specific parameter vectors ${\underset{∼}{\mu }}_{k}$ and ${\underset{∼}{\gamma }}_{k}$, where ${\underset{∼}{\mu }}_{k}=(\mu_{k1},\mu_{k2},\ldots ,\mu_{kI})$, with $\sum_{i=1}^{I}\mu_{ki}=0$, models the pattern of changes in abundance level across treatments and ${\underset{∼}{\gamma }}_{k}=(\gamma_{0k1},\gamma_{1k1},\ldots ,\gamma_{0kI},\gamma_{1kI})$ is the vector modeling *q_kij_* the probabilities of being positive counts for features in cluster *k*. Based on a mixture of Poisson hurdle models, the likelihood given observations of feature *g* can be expressed by $L_{g}=\sum_{k=1}^{K}p_{k}f(\alpha_{gk},{\underset{∼}{\mu }}_{k},{\underset{∼}{\gamma }}_{k}|{\underset{∼}{N}}_{g})$, where ${\underset{∼}{N}}_{g}$ represents the count vector for *g*th feature across all samples, *f* is the probability mass function for Poisson hurdle model (1), and *p_k_* is the mixing proportion corresponding to component *k* with $p_{k}\geq 0$ and $\sum_{k=1}^{K}p_{k}=1$.

The high dimensionality of microbiome data and complex relationships among microbial features makes it nearly impossible to model the dependency among features. In this article, we assume independence among features, but we evaluate the performance of our procedure under more realistic compositional structures among features. With independence assumption, the total likelihood can be expressed by

$$L=\prod_{g}L_{g}=\prod_{g=1}^{G}\sum_{k=1}^{K}p_{k}f(\alpha_{gk},{\underset{∼}{\mu }}_{k},{\underset{∼}{\gamma }}_{k}|{\underset{∼}{N}}_{g}).$$

### 2.2 Poisson hurdle clustering via the EM algorithm

We apply an EM algorithm to obtain parameter estimates and clustering results. The EM algorithm for model-based clustering introduces a latent variable *Z_gk_* as the indicator that feature *g* belongs to cluster *k* for each combination of *g* and *k*. These indicators are treated as missing data, and the conditional expectation $E[Z_{gk}|\underset{∼}{\theta }]$, where $\underset{∼}{\theta }=(p_{k},\alpha_{gk},{\underset{∼}{\mu }}_{k},{\underset{∼}{\gamma }}_{k})$, gives the conditional probability that feature *g* belongs to cluster *k*. EM algorithm proceeds by iteratively calculating conditional expectations (E step) and updating all unknown parameters by maximizing the likelihood function (M step) until convergence. Clustering results are obtained based on the final conditional expectations. EM algorithm is a common approach in model-based clustering (Fraley and Raftery, 2002). Si *et al.* (2014) and Rau *et al.* (2011) implemented EM algorithm in Poisson model-based clustering.

Compared to Poisson models, Poisson hurdle models are much more complicated due to the two-part structure. The total log-likelihood for mixture of Poisson hurdle models involve an extremely high dimension of parameters, and there are no closed-form solutions for the maximum likelihood estimator (MLE) of *μ_ki_* and *α_gk_* in the M step. This poses extra challenges for the EM algorithm. In this article, we propose to utilize a numerical method based on coordinate descent in the M step.

Algorithm 1: EM algorithm for Poisson hurdle clustering.

1. Initialization (*m *=* *1):

Set $p_{k}^{\left(1\right)}=1/K,k=1,\ldots ,K$.

For each cluster *k*, obtain the initial values for parameters: ${\underset{∼}{\gamma }}_{k}^{\left(1\right)},{\underset{∼}{\mu }}_{k}^{\left(1\right)}$ with $\sum_{i=1}^{I}\mu_{ki}^{\left(1\right)}=0$, and ${\underset{∼}{\alpha }}_{k}^{\left(1\right)}$, where ${\underset{∼}{\alpha }}_{k}=(\alpha_{1k},\ldots ,\alpha_{Gk})$.

See Section 2.4 and Supplementary Section S3 for our proposed initialization method.

2. E step:

In the *m*th iteration, calculate the conditional expectation $E[Z_{gk}|{\underset{∼}{\theta }}^{\left(m\right)}]$, denoted as ${\hat{Z}}_{gk}^{\left(m\right)}$ by:

(4) $${\hat{Z}}_{gk}^{\left(m\right)}=\frac{p_{k}^{\left(m\right)}f({\underset{∼}{N}}_{g}|\alpha_{gk}^{\left(m\right)},{\underset{∼}{\mu }}_{k}^{\left(m\right)},{\underset{∼}{\gamma }}_{k}^{\left(m\right)})}{\sum_{l=1}^{K}p_{l}^{\left(m\right)}f({\underset{∼}{N}}_{g}|\alpha_{gl}^{\left(m\right)},{\underset{∼}{\mu }}_{l}^{\left(m\right)},{\underset{∼}{\gamma }}_{l}^{\left(m\right)})}$$

3. M step:

Given ${\hat{Z}}_{gk}^{\left(m\right)}$, the mixing proportion *p_k_* is updated by

$$p_{k}^{\left(m+1\right)}=\frac{\sum_{g}{\hat{Z}}_{gk}^{\left(m\right)}}{G}$$

Maximizing the likelihood function is equivalent to maximizing the following log-likelihood for each cluster *k*, with the constraint $\sum_{i=1}^{I}\mu_{ki}=0$.

$$\begin{array}{c} l_{k}({\underset{∼}{\mu }}_{k},{\underset{∼}{\gamma }}_{k},{\underset{∼}{\alpha }}_{k})=\sum_{g}{\hat{Z}}_{gk}^{\left(m\right)}*\;\text{log}\;f({\underset{∼}{N}}_{g}|{\underset{∼}{\mu }}_{k},{\underset{∼}{\gamma }}_{k},\alpha_{gk})\\ =\sum_{g}{\hat{Z}}_{gk}^{\left(m\right)}*\{\sum_{i,j\in C_{g}}\;\text{log}\;(1-q_{\mathrm{kij}})\\ +\sum_{i,j\notin C_{g}}[\;\text{log}\;q_{\mathrm{kij}}+N_{\mathrm{gij}}\;\text{log}\;\lambda_{\mathrm{gij}}\\ -\lambda_{\mathrm{gij}}-\text{log}(1-e^{-\lambda_{\mathrm{gij}}})]\} \end{array},$$

where $C_{g}=\{i,j:N_{\mathrm{gij}}=0\},\;\lambda_{\mathrm{gij}}=\text{exp}(s_{ij}+\alpha_{gk}+\mu_{ki}),\;q_{\mathrm{kij}}=\frac{1}{1+\text{exp}[-(\gamma_{0ki}+\gamma_{1ki}s_{ij})]}$.We can maximize over the two set of parameters $\gamma_{0ki},\gamma_{1ki}$ and $\alpha_{gk},\mu_{ki}$ separately. But still, there are no closed-form solutions for the maximum likelihood estimate (MLE) of those parameters. Hence, we propose to use a one-step coordinate descent algorithm to obtain numerical solutions for MLEs, which greatly reduce computation. Please see Supplementary Section S2 for more details.

4. Iterate the E step and M step until convergence, i.e. when the change in total likelihood is relatively small.

5. Obtain ${\hat{Z}}_{gk}$ from the last iteration, and assign feature *g* into cluster *k* where $k=\underset{l}{\text{argmax}}{\hat{Z}}_{gl}$.

### 2.3 Simulated annealing modification

As a strictly ascending algorithm, EM algorithm can be trapped in a local maximum. Various methods for adding randomness to help EM algorithm escape from local maximum have been introduced, and simulated annealing (SA) is one of them. The SA algorithm modifies the way to obtain conditional expectation in (3) by introducing a ‘temperature’ $t^{\left(m\right)}>0$ and a ‘cooling rate’ c∈(0,1) as follows:

$$\begin{array}{c} {\tilde{Z}}_{gk}^{\left(m\right)}=\frac{{\left[p_{k}^{\left(m\right)}f({\underset{∼}{N}}_{g}|\alpha_{gk},{\underset{∼}{\mu }}_{k}^{\left(m\right)},{\underset{∼}{\gamma }}_{k}^{\left(m\right)})\right]}^{1/t^{\left(m\right)}}}{\sum_{l=1}^{K}[p_{l}^{\left(m\right)}f({\underset{∼}{N}}_{g}|\alpha_{gl},{\underset{∼}{\mu }}_{l}^{\left(m\right)},{\underset{∼}{\gamma }}_{l}^{\left(m\right)}){]}^{1/t^{\left(m\right)}}},\\ t^{\left(m+1\right)}=c\times t^{\left(m\right)}. \end{array}$$

Given ${\tilde{Z}}_{gk}^{\left(m\right)}$, the SA algorithm clusters each feature *g* into class *k* with multinomial probability ${\tilde{Z}}_{gk}^{\left(m\right)}$ and generates an indicator matrix with entries 0 or 1 that replaces the ${\hat{Z}}_{gk}$ in the M step of the original EM algorithm. This clustering step of SA introduces more randomness (Celeux and Govaert, 1992) that is controlled by the temperature *t*, and larger *t* leads to larger randomness. SA usually starts with a relatively high temperature $t^{\left(0\right)}$ and slowly reduces it to 0 as the algorithm proceeds, and the cooling rate *c* controls the speed of reduction. van Laarhoven and Aarts (1987) recommended $t^{\left(0\right)}=2$ and *c *=* *0.9 which is what we use. Simulation results in Section 3.3 show that SA algorithm yields competitive result compared with the original EM algorithm (Algorithm 1).

### 2.4 Initialization

EM algorithm is an iterative, strictly ascending algorithm whose convergence rate and final results are significantly influenced by the initialization (Biernacki *et al.*, 2003; McLachlan *et al.*, 2008; Melnykov and Melnykov, 2012). Commonly used approaches to initialize the EM algorithm start by picking *K* observations that are far from each other regarding some distance measure, such as (1 − Pearson’s correlation), Euclidean distance (Arthur and Vassilvitskii, 2007), ranked Euclidean distance (Melnykov and Melnykov, 2012) and likelihood ratios (Si *et al.*, 2014), etc. In this article, we propose to use (1−τ) as the distance measure, where *τ* is the Kendall’s *τ* correlation.

We gave a detailed description about the Kendall’s *τ* correlation and our proposed initialization algorithm in Supplementary Section S3. The main idea is that, we first select *K* observations that are well separated measured by (1−τ), and then we obtain MLEs of the model parameters based on the *K* selected observations and use these MLEs as the starting values for our EM algorithm.

In practice, we also recommend utilizing multiple sets of starting values to further avoid being trapped in local maximum, i.e. run the entire algorithm multiple times with different starting values and pick the result with the largest likelihood. Increasing the number of starts tends to provide a better performance at the cost of linearly increasing computation time. In the simulation section, we will show how this affect the clustering results.

### 2.5 Determining number of clusters

In real data analysis, the number of clusters *K* is typically not available and needs to be selected. There are existing methods developed for this purpose, but they do not fit into the scenario we are dealing with. In this article, we propose a hybrid clustering method with the application of likelihood ratio tests to select the number of clusters *K*. The likelihood ratio tests determine the optimal number of *K* by testing, at each step of merging clusters, whether the merging will result in a reduced model that no longer fits data well.

Please refer to Supplementary Section S4 for the discussion of existing methods, the reason why we do not use them, and our complete algorithm for choosing *K*.

## 3 Simulation studies

In this section, we present simulation studies to evaluate the performance of our proposed clustering methods and several existing clustering methods, including Poisson model-based clustering, negative binomial model-based clustering (Si *et al.*, 2014), and the K-means clustering that has been applied to microbiome data (Gloor *et al.*, 2016).

### 3.1 Simulation settings

We consider an experiment with *I *=* *3 treatment groups, *J *=* *5 replicates in each treatment group, and a total of *G *=* *1000 features that belong to *K *=* *7 true clusters. We assume equal mixing proportions of $p_{k}=1/7$. A case of unequal mixing proportion is presented in Supplementary Section S5.

Data are simulated by a zero-inflated negative binomial model which introduces overdispersion, i.e. the variability is more than what is expected by our Poisson model. Each observation, $N_{\mathrm{gij}}\in$ class *k*, is a product of a Bernoulli $\left(q_{\mathrm{kgij}}\right)$ random variable and a negative binomial random variable with mean $E(N_{\mathrm{gij}})=\text{exp}(s_{ij}+\alpha_{g}+\mu_{ki})$ and variance $\left(1+\beta \;\text{exp}(\alpha_{g}))E(N_{\mathrm{gij}}\right)$

For the negative binomial random variable:

1. Overdispersion rate is $\beta \;\text{exp}(\alpha_{g})$, which depends on feature abundance level *α_g_*.
2. The geometric mean abundance levels *α_g’_*s and sequencing depth factors *s_ij_’*s are drawn from a Uniform(0.8,1.2) distribution.
3. *β* controls the overdispersion and ranges from 0 to 0.5. This allows the overdispersion rate to range from 0 to $0.5*e^{1.2}=1.66$, a reasonably large value for overdispersion.
4. The cluster-specific profile across treatment groups, *μ_ki_*, is generated by $\mu_{ki}=\eta_{\mu }\delta_{ki}$ where $\eta_{\mu }$ determines the magnitude of changes across treatments, and larger $\eta_{\mu }$ results in better separation of clusters. ${\underset{∼}{\delta }}_{k}=(\delta_{k1},\delta_{k2},\delta_{k3})$ characterizes the treatment effects in cluster *k* and is generated as follows:

**Table:** 

Cluster *k*	1	2	3	4	5	6	7
$\delta_{k1}$	0	0	1	−1	1	−1	0
$\delta_{k2}$	1	−1	0	0	−1	1	0
$\delta_{k3}$	−1	1	−1	1	0	0	0

Note that the first six clusters cover different abundance profiles across treatments by including all six permutations of three different treatment effects: positive effect (1), no effect (0), and negative effect (−1). The last cluster corresponds to non-differential features whose abundance levels do not change across treatments. Such microbes are typically not of interest but exist in real data and affect the clustering performances.

For the Bernoulli$\left(q_{\mathrm{kgij}}\right)$ random variable that controls zero inflation, we generate it as $q_{\mathrm{gkij}}=\frac{1}{1+\text{exp}[-(\gamma_{0ki}+\gamma_{1ki}s_{ij}+\gamma_{2ki}\alpha_{g})]}$. For each combination of cluster *k* and treatment *i*, we independently draw $\gamma_{1ki},\gamma_{2ki}∼$ Uniform(0,0.5) and set $\gamma_{0ki}$ such that the average zero-inflation rate ${\bar{q}}_{ki}$ in cluster *k* treatment *i* is a specific value in each of the following two scenarios:

1. Set the matrix ${\left\{{\bar{q}}_{ki}\right\}}_{7\times 3}={\left(\begin{array}{ccccccc} 0.9 & 0.9 & 0.3 & 0.6 & 0.3 & 0.6 & 0.5\\ 0.6 & 0.3 & 0.9 & 0.9 & 0.6 & 0.3 & 0.5\\ 0.3 & 0.6 & 0.6 & 0.3 & 0.9 & 0.9 & 0.5 \end{array}\right)}^{T}$.
2. Set ${\bar{q}}_{ki}=\phi \in (0,1]$ for all *k* and *i*.

Note that in the first scenario, all six permutations of high, medium and low zero inflation are present along with a group with equal mean zero-inflation rate (0.5,0.5,0.5) across treatment groups. This is a case where the zero-inflation structure varies significantly among clusters and is more aligned with our model assumptions. In the second scenario, all clusters share the same mean zero-inflation rate with ϕ=1 corresponding to no zero inflation, and the difference among clusters are only reflected through the Poisson mean structure. This is a less desirable circumstance for our method because the zero-inflation rate does not distinguish different clusters. In the main text, we will present simulation results for the second scenario that demonstrate our method performs better than others even in this unfavorable situation. Results for the first scenario are presented in the Supplementary Figure S2.

Finally, after a raw dataset is generated from the above procedure, we do an additional multinomial resampling on each column, with total counts C*G=1000C and probability vector proportional to each column of this raw dataset. This is to mimic the sequencing procedure and generate a compositional dataset with equal column sums. It brings in extra randomness and deviation from our assumed models thus can test the robustness of clustering methods.

We set the default simulation setting with $\beta =0.02,\eta_{\mu }=1,\phi =0.4$, and average sequencing depth (total count/*G*) *C *=* *10. By varying each parameter at a time, we generate data for a variety of different simulation settings. For each simulation setting, 1000 datasets are simulated.

### 3.2 Simulation results

For each simulated dataset, we cluster the 1000 features into seven clusters using five different methods under comparison: (i) Poisson hurdle model-based clustering with EM algorithm (PH-EM), (ii) Poisson hurdle model-based clustering with simulated annealing (PH-SA), (iii) Poisson model-based clustering (MB-Poisson), (iv) negative binomial model-based clustering (MB-NB) and (v) K-means clustering with Euclidean distance (other popular non-model-based methods such as spectral clustering and hierarchical clustering produce similar or worse results and are not presented). The first four model-based methods are applied to the count data, while K-means is applied to the data after centered log ratio (clr) transformation (Aitchison, 1982), as was done by Gloor *et al.* (2016).

The clustering results are evaluated by three criteria: purity, adjusted Rand index (ARI) and normalized mutual information (NMI). All three criteria measure the agreement between clustering results and true clusters used to generate data, within the range of [0,1] with higher values indicating better performance. Purity measures how ‘pure’ the clusters are, i.e. to what extent each resulting cluster contains a single true cluster. Adjusted Rand index (Hubert and Arabie, 1985; Rand, 1971) measures similarity between two partitions (clustering results and true clusters) based on the proportion of pairs of features that are ‘correctly’ assigned. Mutual information (MI) measures the shared information between two partitions. The normalized MI (Strehl and Ghosh, 2002) adjusts the values so that NMI is in [0,1]. See Supplementary Section S6 for definitions with mathematical expressions of all three criteria.

The results from all three criteria are consistent and show the same relative ranking of methods. In the main text, we present results based on NMI. Results on purity and ARI are presented in Supplementary Figure S1. We also evaluated the efficiency of the EM algorithm by checking the computational time and the number of iterations for convergence, which is described in Supplementary Section S7.

#### 3.2.1 Clustering results

**Fig. 1.**
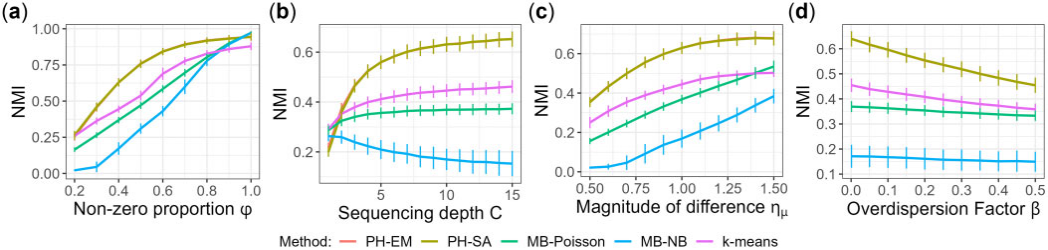
Simulation results for the second simulation scenario. For each setting, we plot the NMI score averaged across the 1000 simulated datasets with the vertical bar representing standard error. The default setting is $\phi =0.4,C=5,\eta_{\mu }=1,\beta =0.02$. Each of (**a–d**) varies one parameter at a time. The curves for PH-EM and PH-SA almost overlap with each other

Figure 1 presents results for a variety of settings of the second simulation scenario when the zero-inflation rates are constant across treatment groups. For all simulation settings, our proposed algorithms, PH-EM and PH-SA are almost indistinguishable from each other, and they are the best performers among all methods under comparison. Figure 1a shows that both PH-EM and PH-SA perform much better than the other methods when zero-inflation rate is between 0.2 and 0.8, a range commonly encountered in real data. When there is no zero inflation at all (ϕ=1), our algorithms still performs similarly to the other two model-based clustering methods that do not model zero inflation and better than K-means. As sequencing depth grows (Fig. 1b), the magnitude of treatment differences increases (Fig. 1c) or the level of fluctuation decreases (Fig. 1d), most methods tend to perform better. When sequencing depth is low, our methods are among the top-performing methods. For all other settings (Fig. 1b–d), our methods are the best with obvious advantage over the other methods. These results show the consistency and robustness of the Poisson hurdle clustering algorithms. Results from the first simulation scenario (different zero-inflation structure among clusters) presented in Supplementary Figure S2 give the same conclusion.

#### 3.2.2 Evaluation of initialization

**Fig. 2.**
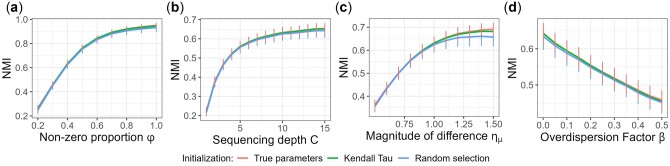
Comparison of initialization methods. For each setting, we plot the NMI score averaged across the 1000 simulated datasets, with the vertical bar representing standard deviation

We also compare three initialization methods: (i) using true parameters, (ii) using the MLEs based on *K* randomly selected observations and (iii) using the Kendall’s *τ* based initialization algorithm we propose in Section 2.4. As shown by the NMI results in Figure 2, our proposed initialization method uniformly outperforms random selection and is close to the result using true parameters which is the best we can get in simulation but not available in real data analysis. Results of purity and ARI as performance measure are in Supplementary Figure S3 and give the same conclusion.

In Supplementary Figure S4, we present the results of using different number of starts when we utilize multiple starting strategy. When we use 5 starting values, we get significant improvement in performance, while increasing to 10 starting values does not seem to have an additional notable effect. Therefore we choose 5 starts in all simulation studies.

#### 3.2.3 Determining the number of clusters

In Section 2.5 and Supplementary Section 4, we describe a method for determining the number of clusters. Here, we evaluate this method using 2000 datasets generated from the default simulation setting where the true number of clusters is *K *=* *7. Table 1 shows the proportions of those datasets being identified with certain number clusters using four different methods: our proposed Hybrid method, Akaike information criterion, Bayesian information criterion and Gap statistic (Tibshirani *et al.*, 2001). We can see that while those three methods tend to greatly underestimate the number of clusters, results from our hybrid method remain close to the true value *K *=* *7.

**Table 1.** The percentage of 2000 simulated datasets for each number of clusters chosen by four different methods

No. of clusters (%)	1	2	3	4	5	6	7	8
Hybrid	0	0	0	0	0.60	11.45	86.75	1.20
AIC	0	0	94.60	4.30	1.05	0.05	0	0
BIC	0	0.7	98.95	0.35	0	0	0	0
Gap	91.65	3.75	3.85	0.40	0.20	0.15	0	0

*Note*: The true number of clusters is 7.

## 4 Real data analysis

A microbiome study was carried out in Nebraska where sorghum plants of the genotype Grassl were grown with two varying nitrogen levels (low/high). Rhizosphere microbiome samples were collected on four different dates throughout the growing season between June and September of 2017 and analyzed by 16S rRNA amplicon sequencing (Qi *et al.*, 2021). Applying the persistence method (Shade and Handelsman, 2012) to identify core ASVs among the eight treatments, we obtained a subset of 449 ASVs with an average read count of 44.21, and 38% of counts in this subset are 0. Our proposed method for determining the number of clusters chose *K *=* *30.

**Fig. 3.**
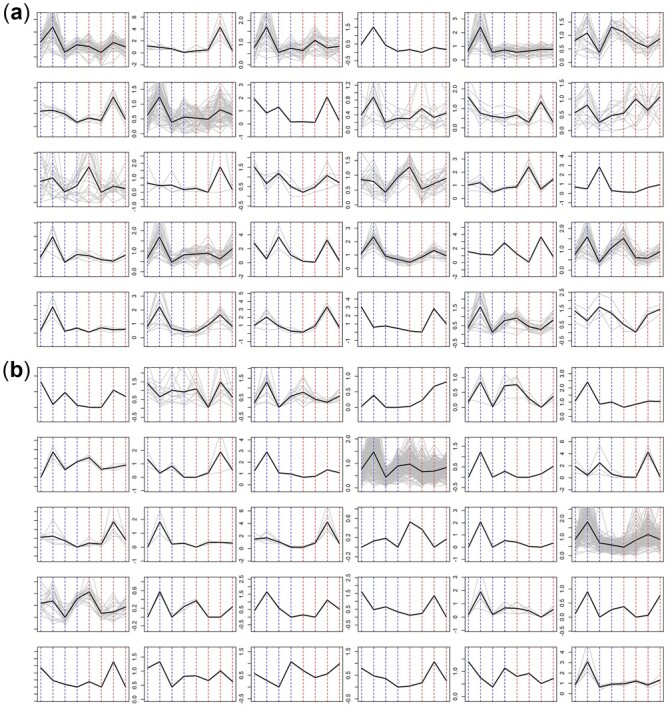
Results from sorghum data analysis based on (**a**) PH-EM algorithm and (**b**) model-based NB clustering. Each subplot corresponds to one cluster, with x-axis corresponding to the 8 treatment groups (two nitrogen levels with four chronically ordered dates, 1–4 correspond to high nitrogen, 5–8 correspond to low nitrogen) and y-axis corresponding to the abundance profile. Each grey line corresponds to the abundance profile estimated by the method of moments for an ASV, and the black line plots the geometric mean within each cluster. For each method, clusters will be referred to as Cluster No. 1–6 for the first row, 7–12 for the second row, …, and 25–30 for the last row

We applied all four model-based clustering algorithms to the original count data and also applied the K-means clustering to the clr-transformed data to group the 449 ASVs into 30 clusters. Figure 3 plots the abundance profiles of all 30 clusters identified by our PH-EM method (Fig. 3a) and model-based NB clustering method (Fig. 3b), respectively. The Poisson model-based clustering produced worse results than the NB model-based method and is not shown here. Results for the PH-SA method and K-means are presented in Supplementary Figure S5 and yield similar conclusions.

As shown in Figure 3a, the PH-EM method resulted in better separation of different clusters and more similar profiles within each cluster. In contrast, in Figure 3b, model-based NB clustering clusters over 80% of ASVs into 2 huge clusters, and 28 small clusters of which 15 are singletons. To parse finer-scale relationships between taxa in those two huge clusters, further clustering would be needed, complicating interpretation and placing additional bioinformatic burden on the researcher. This is not the case with our method, making it more useful for researchers looking to gain exploratory insight into the biological or ecological structure of microbial communities.

Our method revealed several bacterial clusters whose abundance was sensitive to plant developmental stage and nitrogen concentration. For example, PH-EM clustering revealed several clusters consisting of plant-growth-promoting taxa (e.g. *Pseudomonas*, *Sphingomonas*, *Rhizobium*, *Arthrobacter* and *Streptomyces*) whose abundance remained relatively stable under high nitrogen, but increased dramatically under low nitrogen (Fig. 3a, clusters 2, 7, 9, 11, 14 and 27). These patterns suggest that under low nitrogen a putative guild of nitrogen-fixing taxa is selected for, at least partly driven by root exudation later in sorghum development. These results were corroborated by our analysis of amplicon sequences from 2016, as well as metagenomes assembled from both 2016 and 2017 samples that showed a significant increase in nitrogen-fixing genes under low nitrogen (Supplementary Fig. S6). Similar patterns have been identified in other studies of the sorghum rhizosphere (Hara *et al.* 2019; Lopes *et al.* 2021; Yu *et al.* 2011; Wu *et al.*, 2021). Interestingly, this nitrogen-fixing guild seems to be preceded by other putatively plant-growth-promoting taxa, such as *Massilia* and *Bacillus* (Clusters 12 and 17), whose abundances were also higher under low nitrogen. Importantly, these patterns were masked in the model-based NB clusters 10 and 18 whose average trends suggest the opposite: low abundance under low nitrogen and high abundance under high nitrogen. Based on these findings, we believe our clustering method could be particularly useful for parsing dynamic ecological relationships from datasets consisting of times series and/or many treatments.

**Fig. 4.**
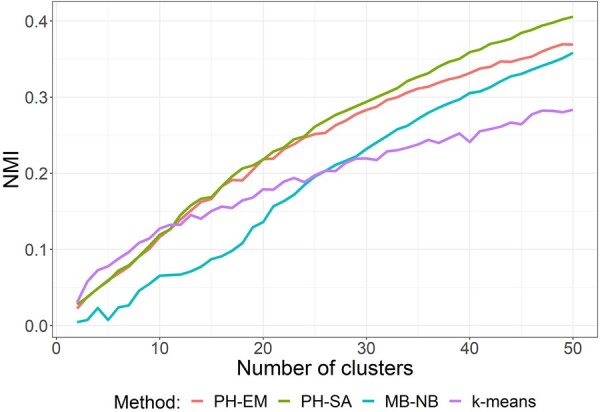
Clustering results for the rhizosphere microbiome dataset. Clustering results obtained by PH-EM, PH-SA, model-based negative binomial clustering (MB-NB), and K-means are compared with genera categories for each number of clusters *K*, ranging from 2 to 50. The NMI values shown are averages from 100 clustering results at each *K*

To provide a quantitative evaluation of the clustering results, we measured the concordance between clustering results and taxonomic categories at the genus level. With the number of clusters, *K*, ranging from 2 to 50, we performed cluster analysis with different methods and calculated NMI based on the concordance between each clustering result and genera. Figure 4 shows that both PH-EM and PH-SA produced higher NMI values than K-means and model-based NB clustering for *K* larger than 7. When *K* is small (2–7), the model-based NB method gave slightly higher NMI values than our algorithms. Thus, our method outperformed other clustering methods when it came to clustering microbes based on microbial taxonomy, a proxy for ecological similarity. It is important to acknowledge that we did not expect our model to find perfect agreement between taxonomy and abundance (i.e. NMI values near 1). This is because microbiomes represent complex communities that can display large variation across individuals which cannot be explained by deterministic assembly mechanisms alone. The absence of perfect agreement between taxonomy and abundance could represent an alternative perspective: the combined influence of deterministic and stochastic assembly factors characteristic of competitive lottery models or priority effects (Sale, 1979). In situations where a particular niche space can support only a single species, stochastic dispersal followed by high competition among related species can result in only one ‘winning’ species—the identity of which can vary independently of any niche effect (Lee *et al.* 2013; Peay *et al.* 2012; Verster and Borenstein 2018). This could explain why ASVs of a particular genus group into more than one cluster.

For example, we found that *Pseudomonas* sp. from the sorghum rhizosphere grouped into 12 distinct clusters as compared to just 4 clusters with the model-based NB method. Our amplicon datasets from 2015 suggested that the sorghum rhizosphere exhibits a significant reduction in community diversity due to a ‘bloom’ of a diverse collection of *Pseudomonas* ASVs. Further analysis of isolate genomes and rhizosphere metagenomes from these samples revealed that this community represents several distinct *Pseudomonas* lineages that vary in their functional capacity especially regarding their commensal or pathogenic relationship with the host plant (Chiniquy *et al.*, 2021). This diversity resulted in subtle but distinct abundance patterns between lineages. As mentioned above, our PH-EM method captured these abundance patterns in our 2017 dataset, while the model-based NB method failed to identify this heterogeneity, instead clustering most of these *Pseudomonas* sp. into a single large cluster, 18. Further, the PH-EM method confirmed that the abundances of many (although not all) of these *Pseudomonas* sp. are significantly higher under low nitrogen as compared to high nitrogen (Fig. 3a, clusters 2, 7, 14, 23 and 28). These patterns are undiscernible from the model-based NB results which clustered Pseudomonas ASVs with contrasting high and low nitrogen abundance patterns into the same cluster (Fig. 3b, cluster 18). Thus, in addition to identifying niche effects in microbial communities, our clustering method may also help researchers hone in on more complex ecological relationships that can be isolated, tested and confirmed via manipulative studies.

## 5 Discussion

Many features of microbiome data are zero-inflated while others do not exhibit zero inflation. Traditional clustering methods do not consider such characteristics. In this article, we model microbiome data with Poisson hurdle distributions that can fit features with or without zero inflation. To cluster features, we fit a mixture Poisson hurdle model with an EM algorithm (PH-EM) or another algorithm with a SA modification (PH-SA). Both algorithms have superior performances over other algorithms in both simulation studies and real data analysis. We also propose an initialization method and a method for determining optimal number of clusters, which are shown to be effective in our simulation studies. Compared with non-model-based clustering algorithms such as K-means method, our clustering algorithms also provide the uncertainties of the clustering results.

We also considered further extending our algorithms to negative binomial hurdle distributions to accommodate potential over-dispersion that may exist in real dataset. We decided not to do so, due to the inefficiency in estimating the overdispersion parameter. Detailed explanations and simulation results are provided in Supplementary Section S8.

## Supplementary Material

btac782_Supplementary_Data

## Data Availability

All datasets used in this article are publicly and freely available at the JGI Genome Portal.

## References

[btac782-B1] Aitchison J. (1982) The statistical analysis of compositional data. J. R. Stat. Soc. B, 44, 139–160.

[btac782-B2] Arthur D. , Vassilvitskii S. (2007) K-means++: the advantages of careful seeding. In *Proceedings of the Eighteenth Annual ACM-SIAM Symposium on Discrete Algorithms, SODA 2007, New Orleans, Louisiana, USA, January 7-9, 2007*, Vol. 8, pp. 1027–1035. 10.1145/1283383.1283494.

[btac782-B3] Badri M. et al (2020) Shrinkage improves estimation of microbial associations under different normalization methods. NAR Genom. Bioinform., 2, lqaa100. 33575644 10.1093/nargab/lqaa100 PMC7745771

[btac782-B4] Biernacki C. et al (2003) Choosing starting values for the em algorithm for getting the highest likelihood in multivariate Gaussian mixture models. Comput. Stat. Data Anal., 41, 561–575.

[btac782-B5] Bullard J.H. et al (2010) Evaluation of statistical methods for normalization and differential expression in mRNA-seq experiments. BMC Bioinformatics, 11, 94. 20167110 10.1186/1471-2105-11-94 PMC2838869

[btac782-B6] Casero D. et al (2017) Space-type radiation induces multimodal responses in the mouse gut microbiome and metabolome. Microbiome, 5, 105. 28821301 10.1186/s40168-017-0325-z PMC5563039

[btac782-B7] Celeux G. , Govaert G. (1992) A classification EM algorithm for clustering and two stochastic versions. Comput. Stat. Data Anal., 14, 315–332.

[btac782-B8] Chiniquy D. et al (2021) Microbial community field surveys reveal abundant pseudomonas population in sorghum rhizosphere composed of many closely related phylotypes. Front. Microbiol., 12, 598180. 33767674 10.3389/fmicb.2021.598180 PMC7985074

[btac782-B9] Cragg J.G. (1971) Some statistical models for limited dependent variables with application to the demand for durable goods. Econometrica, 39, 829–844.

[btac782-B10] Cullen C.M. et al (2020) Emerging priorities for microbiome research. Front. Microbiol., 11, 136. 32140140 10.3389/fmicb.2020.00136 PMC7042322

[btac782-B11] Fraley C. , Raftery A.E. (2002) Model-based clustering, discriminant analysis, and density estimation. J. Am. Stat. Assoc., 97, 611–631.

[btac782-B12] Gloor G.B. et al (2016) It’s all relative: analyzing microbiome data as compositions. Ann. Epidemiol., 26, 322–329. 27143475 10.1016/j.annepidem.2016.03.003

[btac782-B13] Gloor G.B. et al (2017) Microbiome datasets are compositional: and this is not optional. Front. Microbiol., 8, 2224. 29187837 10.3389/fmicb.2017.02224 PMC5695134

[btac782-B14] Hara S. et al (2019) Identification of nitrogen-fixing bradyrhizobium associated with roots of field-grown sorghum by metagenome and proteome analyses. Front. Microbiol., 10, 407. 30915047 10.3389/fmicb.2019.00407 PMC6422874

[btac782-B15] Hilbe J.M. (2011) Negative Binomial Regression, 2nd edn. Cambridge University Press, Cambridge, England.

[btac782-B16] Hubert L. , Arabie P. (1985) Comparing partitions. J. Classif., 2, 193–218.

[btac782-B17] Lee S.M. et al (2013) Bacterial colonization factors control specificity and stability of the gut microbiota. Nature, 501, 426–429. 23955152 10.1038/nature12447 PMC3893107

[btac782-B18] Lonèar-Turukalo T. et al (2019) Clustering of microbiome data: evaluation of ensemble design approaches. In: *IEEE EUROCON 2019 -18th International Conference on Smart Technologies*, Novi Sad, Serbia. pp. 1–6.

[btac782-B19] Lopes L.D. et al (2021) Sweet sorghum genotypes tolerant and sensitive to nitrogen stress select distinct root endosphere and rhizosphere bacterial communities. Microorganisms, 9, 1329. 34207412 10.3390/microorganisms9061329 PMC8234256

[btac782-B20] McLachlan G. et al (2008) The EM Algorithm and Extensions. Wiley Series in Probability and Statistics. Wiley.

[btac782-B21] McMurdie P.J. , Holmes S. (2014) Waste not, want not: why rarefying microbiome data is inadmissible. PLoS Comput. Biol., 10, e1003531. 24699258 10.1371/journal.pcbi.1003531 PMC3974642

[btac782-B22] Melnykov V. , Melnykov I. (2012) Initializing the EM algorithm in Gaussian mixture models with an unknown number of components. Comput. Stat. Data Anal., 56, 1381–1395.

[btac782-B23] Peay K.G. et al (2012) Phylogenetic relatedness predicts priority effects in nectar yeast communities. Proc. Biol. Sci., 279, 749–758. 21775330 10.1098/rspb.2011.1230 PMC3248732

[btac782-B24] Poretsky R. et al (2014) Strengths and limitations of 16s rRNA gene amplicon sequencing in revealing temporal microbial community dynamics. PLoS One, 9, e93827. 24714158 10.1371/journal.pone.0093827 PMC3979728

[btac782-B25] Qi M. et al (2021) Identification of beneficial and detrimental bacteria that impact sorghum responses to drought using multi-scale and multi-system microbiome comparisons. *ISME J.,* **16**, 1957–1969*.* 10.1038/s41396-022-01245-4 PMC9296637 35523959

[btac782-B26] Rand W.M. (1971) Objective criteria for the evaluation of clustering methods. J. Am. Stat. Assoc., 66, 846–850.

[btac782-B27] Rau A. et al (2011) Clustering high-throughput sequencing data with Poisson mixture models. 10.1093/bioinformatics/btu845 25563332

[btac782-B28] Sale P.F. (1979) Recruitment, loss and coexistence in a guild of territorial coral reef fishes. Oecologia, 42, 159–177. 28309658 10.1007/BF00344855

[btac782-B29] Shade A. , Handelsman J. (2012) Beyond the Venn diagram: the hunt for a core microbiome. Environ. Microbiol., 14, 4–12. 22004523 10.1111/j.1462-2920.2011.02585.x

[btac782-B30] Si Y. et al (2014) Model-based clustering for RNA-seq data. Bioinformatics, 30, 197–205. 24191069 10.1093/bioinformatics/btt632

[btac782-B31] Strehl A. , Ghosh J. (2002) Cluster ensembles - a knowledge reuse framework for combining multiple partitions. J. Mach. Learn. Res., 3, 583–617.

[btac782-B32] Tibshirani R. et al (2001) Estimating the number of clusters in a data set via the gap statistic. J. R. Stat. Soc. B, 63, 411–423.

[btac782-B33] van Laarhoven P.J.M. , Aarts E.H.L. (1987) Simulated Annealing. Springer, Netherlands, Dordrecht, pp. 7–15.

[btac782-B34] Verster A.J. , Borenstein E. (2018) Competitive lottery-based assembly of selected clades in the human gut microbiome. Microbiome, 6, 186. 30340536 10.1186/s40168-018-0571-8 PMC6195700

[btac782-B35] Weiss S. et al (2017) Normalization and microbial differential abundance strategies depend upon data characteristics. Microbiome, 5, 27. 28253908 10.1186/s40168-017-0237-y PMC5335496

[btac782-B36] Wu A.-L. et al (2021) Sorghum rhizosphere effects reduced soil bacterial diversity by recruiting specific bacterial species under low nitrogen stress. Sci. Total Environ., 770, 144742. 33736399 10.1016/j.scitotenv.2020.144742

[btac782-B37] Xu L. et al (2015) Assessment and selection of competing models for zero-inflated microbiome data. PLoS One, 10, e0129606. 26148172 10.1371/journal.pone.0129606 PMC4493133

[btac782-B38] Yeung K.Y. et al (2001) Model-based clustering and data transformations for gene expression data. Bioinformatics, 17, 977–987. 11673243 10.1093/bioinformatics/17.10.977

[btac782-B39] Yu H. et al (2011) Complete genome sequence of the nitrogen-fixing and rhizosphere-associated bacterium *Pseudomonas stutzeri* strain dsm4166. J. Bacteriol., 193, 3422–3423. 21515765 10.1128/JB.05039-11 PMC3133286

[btac782-B40] Zhang Y. et al (2017) Multi-view clustering of microbiome samples by robust similarity network fusion and spectral clustering. IEEE/ACM Trans. Comput. Biol. Bioinform., 14, 264–271. 26513798 10.1109/TCBB.2015.2474387

