# Supplementary material for: Poisson hurdle model-based method for clustering microbiome features
Source: Bioinformatics. 2022 Dec 5;39(1):btac782. doi: 10.1093/bioinformatics/btac782 (PMC9825753; doi:10.1093/bioinformatics/btac782)
Supplement: btac782_Supplementary_Data [file btac782_supplementary_data.zip › PHcluster_final_supplementary.pdf]

Supplementary Materials for

"Poisson hurdle model-based method for clustering microbiome features."

Zhili Qiao, Elle Barnes, Susannah Tringe, Daniel P. Schachtman, and Peng Liu

Contents

1 Clustering Features Using Abundance Levels or Treatment Effects? 3

2 Coordinate Descent Algorithm for Updating Parameters 5

3 Initialization Algorithm 7

4 Determining the Number of Clusters 7

5 Simulation Results for Unequal Cluster Size 9

6 Evaluation Criteria 11

7 Convergence of the EM algorithm 12

8 Extension of Model-based Negative Binomial Hurdle Model 13

9 Supplementary Figure 1 16

10 Supplementary Figure 2 17

11 Supplementary Figure 3 18

12 Supplementary Figure 4 19

13 Supplementary Figure 5 20

14 Supplementary Figure 6 21

1 Clustering Features Using Abundance Levels or Treatment Effects?

In Section 2 of the main text, we present model (1) which specifies that features in the same cluster share the same treatment effects (μ_{ki}) with possibly different geometric means (α_{gk}) across features in the same cluster. With model (1), we can cluster treatment effects, i.e., changes in abundance levels across treatments. This model has a high-dimension of parameters because α_{gk} is in the dimension of $G * K$ where the number of features G is typically large.

Instead of clustering according to treatment effects, we can also cluster features according to their abundance levels by assuming both the same $\alpha_{gk} = \alpha_k$ and the same treatment effects (μ_{ki}) for all features in the same cluster. This α_k model is a reduced model compared to model (1) because feature-wise difference is ignored within the same cluster. It assumes that the abundance levels also contain information about clusters.

These two model forms (α_{gk}/α_k) focus on different targets:

- The α_{gk} model in the main text aims at clustering **relative** abundance profiles of the features because our model clusters features according to treatment effects (μ_{ki}'s), i.e., how feature abundances vary among treatment groups **after** extracting mean abundance levels;
- The α_k model focuses more on the **actual** abundance. Features with similar absolute mean abundance levels are more likely to be clustered together, despite that they might have different patterns across treatments.

Both models have meaningful biological interpretation and may lead to quite different clustering results, researchers may choose between these two models based on their research question or interest.

Next we present modifications on modeling and algorithm for the second case, the α_k model.

Recall that log Poisson mean expression in Section 2 of main text is $\log(\lambda_{kgij}) = s_{ij} + \alpha_{gk} + \mu_{ki}$, with constraint $\sum_i \mu_{ki} = 0$, for all k. In the α_k model it reduces to $\log(\lambda_{kgij}) = s_{ij} + \alpha_k + \mu_{ki} = s_{ij} + \tilde{\mu}_{ki}$ with unconstrained $\tilde{\mu}_{ki} = \alpha_k + \mu_{ki}$. The number of parameters we need to optimize over then reduced from $K(G+3I-1)$ to $3KI$, with additional $2KI$ parameters in estimating $\gamma_{0ki}, \gamma_{1ki}$'s for the zero probabilities.

In the M step of the EM algorithm (see Section 2 in the Supplementary Materials), we can skip the optimization step of α's and only optimize over μ's. The M step then reduces to the maximization problem of $K \times I$ separate univariate functions, which dramatically accelerates computation (within the same number of iterations).

Following the similar settings as in Section 3.1 of the main text, we construct an illustrative simulation study to show the difference between these two models. Let α_g denote the mean abundance level for feature g when generating datasets:

- Simulation setting 1: Simulate 1000 datasets with $\alpha_g \overset{iid}{\sim} Uniform(0, 1.2), g = 1, 2, \cdots, G$ for each average sequencing depths $C = 1, 2, \cdots, 15$;

- Simulation setting 2: Simulate 1000 datasets with $\alpha_{g|g\in C_k} = \alpha_k \overset{iid}{\sim} Uniform(0, 1.2), g = 1, 2, \cdots, G, k = 1, 2, \cdots, K$ for each average sequencing depths $C = 1, 2, \cdots, 15$. Here $g \in C_k$ represents feature g in cluster k.

Other parameter settings are the same as our default setting, see Supplementary Figure 1.

Simulation setting 1 focuses on clustering difference in relative abundance among treatments, where α_g are generated randomly and are considered not effective in determining clusters. Setting 2 has mean abundance levels α_k vary only across clusters, thus they also contain information about cluster assignments.

In each setting, we perform the Poisson hurdle EM clustering (*PH-EM*) with α_{gk} and α_k model. The average performance (Purity, ARI or NMI) across these 1000 datasets are shown in the following figure. In each plot, the red line corresponds to clustering performance using α_{gk} model, blue line corresponds to the one using α_k:

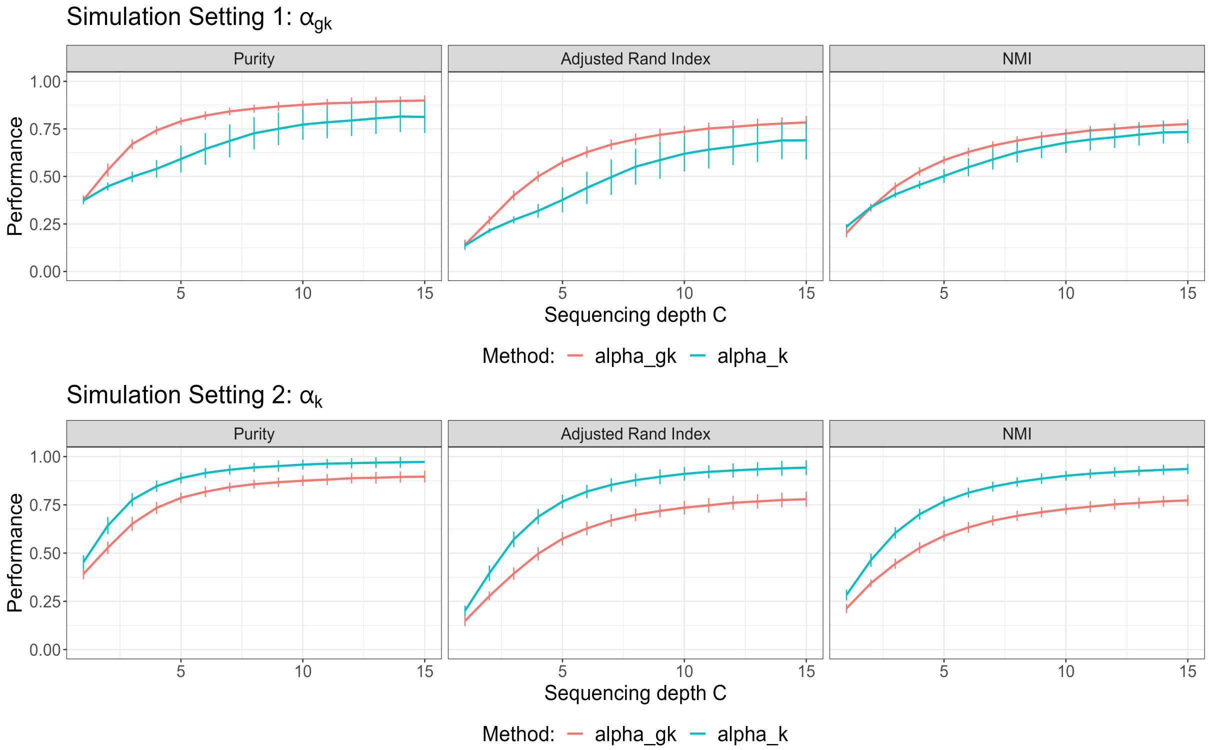

Comparison of the two models under different simulation settings.

As shown in the figure, the method that matches the data generation performs better than the other one. In general, the α_{gk} model (red curve) has consistent performance across the two settings in terms of the values of evaluation criteria, while performance of α_k model (blue curve) differs a lot. In setting 1 the α_k model has less satisfying results. This is reasonable as it "falsely" takes into account the mean abundance levels of each feature when doing clustering, which are in fact generated completely at random. On the contrary, in setting 2 when the mean abundance level does contain information about clusters, α_k model has much higher accuracy than α_{gk} model.

We implemented both models in our R package *PHclust*, with argument

$$absolute = TRUE/FALSE$$

(*FALSE* by default, corresponding to the α_{gk} model). Users may decide by themselves which one fits better into their research question.

2 Coordinate Descent Algorithm for Updating Parameters

In Section 2.2 of the main text, we present our EM algorithm for Poisson hurdle clustering. Here we present more details for the M-step of the EM algorithm.

For each cluster k, the log-likelihood is:

$$\begin{aligned} l_k(\underset{\sim}{\mu}_k, \underset{\sim}{\gamma}_k, \underset{\sim}{\alpha}_k) &= \sum_g \hat{Z}_{gk}^{(m)} * \{ \sum_{i,j \in C_g} \log(1 - q_{kij}) + \sum_{i,j \notin C_g} [\log q_{kij} + N_{gij} \log \lambda_{gij} - \lambda_{gij} - \log(1 - e^{-\lambda_{gij}})]\} \\ &\equiv \sum_{i=1,\ldots,I} l_{1ki}(\underset{\sim}{\gamma}_{ki}) + l_{2k}(\underset{\sim}{\mu}_k, \underset{\sim}{\alpha}_k) \end{aligned}$$

With

$$\begin{aligned} l_{1ki}(\underset{\sim}{\gamma}_{ki}) &= \sum_g \hat{Z}_{gk}^{(m)} \{ \sum_j [\mathbb{1}(N_{gij} = 0) \log(1 - q_{kij}) + \mathbb{1}(N_{gij} > 0) \log q_{kij}]\} \\ &= \sum_g \hat{Z}_{gk}^{(m)} \{ \sum_j [\mathbb{1}(N_{gij} = 0) \log(\frac{1}{1 + \exp[\gamma_{0ki} + \gamma_{1ki} s_{ij}]}) + \mathbb{1}(N_{gij} > 0) \log(\frac{\exp[\gamma_{0ki} + \gamma_{1ki} s_{ij}]}{1 + \exp[\gamma_{0ki} + \gamma_{1ki} s_{ij}]})]\} \\ &= \sum_g \hat{Z}_{gk}^{(m)} \sum_j [-\log(1 + \exp(\gamma_{0ki} + \gamma_{1ki} s_{ij})) + (\gamma_{0ki} + \gamma_{1ki} s_{ij}) \mathbb{1}(N_{gij} > 0)] \\ l_{2k}(\underset{\sim}{\mu}_k, \underset{\sim}{\alpha}_k) &= \sum_g \hat{Z}_{gk}^{(m)} \{ \sum_{i,j \notin C_g} [N_{gij} \log \lambda_{gij} - \lambda_{gij} - \log(1 - e^{-\lambda_{gij}})]\} \end{aligned}$$

with constraint $\sum_i \mu_{ki} = 0$, where $C_g = \{i, j : N_{gij} = 0\}$, $\lambda_{gij} = \exp(s_{ij} + \alpha_{gk} + \mu_{ki})$.

To maximize the total log likelihood, we first deal with the part $l_{1ki}(\underset{\sim}{q}_{ki})$. As an optimization problem with two parameters $\gamma_{0ki}, \gamma_{1ki}$, we utilize a simple coordinate descent algorithm. The coordinate descent algorithm seeks to maximize a function in different marginal directions, by varying each parameter at a time while holding all other parameters unchanged. Here we do it with two steps. For each combination of cluster k and treatment i, do the following:

1. Treating γ_{1ki} as constant, optimize l_{1ki} by univariate gradient descent, with

$$\frac{\partial l_{1ki}}{\partial \gamma_{0ki}} = \sum_g \hat{Z}_{gk}^{(m)} \sum_j [\mathbb{1}(N_{gij} > 0) - \frac{\exp(\gamma_{0ki} + \gamma_{1ki} s_{ij})}{1 + \exp(\gamma_{0ki} + \gamma_{1ki} s_{ij})}]$$

2. Treating γ_{0ki} as constant, optimize l_{1ki} by univariate gradient descent, with

$$\frac{\partial l_{1ki}}{\partial \gamma_{1ki}} = \sum_g \hat{Z}_{gk}^{(m)} \sum_j s_{ij}[\mathbb{1}(N_{gij} > 0) - \frac{\exp(\gamma_{0ki} + \gamma_{1ki} s_{ij})}{1 + \exp(\gamma_{0ki} + \gamma_{1ki} s_{ij})}]$$

This similar idea applies to the other part $l_{2k}(\underset{\sim}{\mu}_k, \underset{\sim}{\alpha}_k)$, but with slightly more complex computation:

$$\begin{aligned} l_{2k}(\underset{\sim}{\mu}_k, \underset{\sim}{\alpha}_k) &= \sum_g \hat{Z}_{gk}^{(m)} * \{ \sum_{i,j \notin C_g} [N_{gij} \log \lambda_{gij} - \lambda_{gij} - \log(1 - e^{-\lambda_{gij}})]\} \\ &= \sum_g \sum_i \sum_j \{\hat{Z}_{gk}^{(m)} * \mathbb{1}(i, j \notin C_g)[N_{gij} \log \lambda_{gij} - \lambda_{gij} - \log(1 - e^{-\lambda_{gij}})]\} \\ &\equiv \sum_g \sum_i h_{gi}(\alpha_{gk} + \mu_{ki}) \qquad (*) \end{aligned}$$

Where the last step comes from the fact that λ_{gij} is a function of $(\alpha_{gk} + \mu_{ki})$.

Claim 2.1. *The constrained optimization* $\max_{\sum_i \mu_{ki}=0} l_{2k}(\underset{\sim}{\mu}_k, \underset{\sim}{\alpha}_k)$ *is equivalent to unconstrained optimization* $\max l_{2k}(\underset{\sim}{\mu}_k, \underset{\sim}{\alpha}_k)$

Proof. For any unconstrained global/local maximum $l_k(\underset{\sim}{\mu}_k^*, \underset{\sim}{\alpha}_k^*)$ and corresponding $(\mu_{ki}^*, \alpha_{gk}^*)$'s, we can always reformulate it into $l_k(\underset{\sim}{\mu}_k^*, \underset{\sim}{\alpha}_k^*) = \sum_g \sum_i h_{gi}(\alpha_{gk}^* + \mu_{ki}^*) = \sum_g \sum_i h_{gi}(\alpha_{gk}^* + \bar{\mu}_{k\cdot}^* + \mu_{ki}^* - \bar{\mu}_{k\cdot}^*)$ and substitute $(\alpha_{gk}, \mu_{ki}) = (\alpha_{gk}^* + \bar{\mu}_{k\cdot}^*, \mu_{ki}^* - \bar{\mu}_{k\cdot}^*)$, which satisfies $\sum_i \mu_{ki} = 0$. □

However, it is still impossible for us to find a closed-form solution for this unconstrained maximization problem of μ_{ki} and α_{gk}'s, and we have to rely on numerical methods. Because λ_{gij} is a function of $\alpha_{gk} + \mu_{ki}$ for each combination of g and i, we proposed to use a **grouped coordinate descent** algorithm with two groups of variables, one group includes all μ_{ki} and the other group includes all α_{gk}. Below is one step of this algorithm:

- Given all $\alpha_{gk}, g = 1, \cdots, G$, log-likelihood in expression (*) above can be further simplified as

$$\sum_i [\sum_g h_{gi}(\alpha_{gk} + \mu_{ki})] \equiv \sum_i h_i(\mu_{ki})$$

  where $h_i(\mu_{ki}) = \sum_g h_{gi}(\alpha_{gk} + \mu_{ki})$ is a univariate function of μ_{ki}. Then univariate gradient descent can be used to finding every single μ_{ki} that maximizes the likelihood function.

- Similarly, given all $\mu_{ki}, i = 1, \cdots, I$, we can write $l_{2k}(\underset{\sim}{\alpha}_k) = \sum_g h_g(\alpha_{gk})$ and get α_{gk} that maximizes this likelihood function.

Usually a general coordinate descent algorithm will repeat the above procedures until convergence. Considering the computational complexity in our case, we only run one step of such procedure in each round of EM iteration. Our simulations suggests that this is adequate for clustering.

3 Initialization Algorithm

In this section, we give an introduction about the Kendall's τ correlation, explain why we use it, and describe in details our initialization algorithm proposed in Section 2.4 of the main text.

The Kendall's τ correlation is a nonparametric correlation measure calculated based on the numbers of concordant pairs and discordant pairs in two numerical vectors of same length. Its formula is given by

$$\tau = \frac{(\#\ of\ concordant\ pairs) - (\#\ of\ discordant\ pairs)}{\#\ of\ pairs}$$

For the highly right-skewed microbiome count data with excessive zeros, Kendall's τ is a more robust measure of correlation compared with Pearson's correlation. It has also been shown that Kendall's τ is preferred over Spearman's correlation with respect to robustness and efficiency (Croux and Dehon, 2010).

Below is the details of our initialization algorithm proposed in Section 2.4 of the main text:

Algorithm 2: Initialization based on Kendall's τ correlation

1. Randomly pick one feature as the starting point;
2. In the kth step where k features $M_k = \{m_1, m_2, \cdots, m_k\}$ have been chosen, pick the feature in the remaining $(G-k)$ features which has the largest total dissimilarity towards those k features. Here the dissimilarity between two features is measured by $(1-\tau)$, where τ is the Kendall's Tau correlation. Add this feature into M_k to get M_{k+1};
3. Repeat step 2 until K features $M_K = (m_1, \cdots, m_K)$ are chosen;
4. Obtain the MLE $\underset{\sim}{\gamma}_k$ and $\underset{\sim}{\mu}_k$ from each single m_k, and use these MLEs as the initial values $\underset{\sim}{\gamma}_k^{(1)}$ and $\underset{\sim}{\mu}_k^{(1)}$. Obtain the initial values $\alpha_{gk}^{(1)}, g = 1, \cdots, G, k = 1, \cdots, K$ by maximizing $f(\underset{\sim}{N}_g | \alpha_{gk}, \underset{\sim}{\mu}_k^{(1)}, \underset{\sim}{\gamma}_k^{(1)})$ for all combinations of g and k.

4 Determining the Number of Clusters

Determining the number of clusters K is typically a big issue in real data applications. For non-model based clustering methods such as K-means and hierarchical clustering, usually distance-based methods are used to choose optimal K. Popular choices include the elbow method, the Silhouette value (Rousseeuw, 1987), and the gap statistic (Tibshirani *et al.*, 2001). However, this type of methods won't work well in model-based clustering algorithms. Model-based clustering assigns data points to clusters based on its likelihood under different mixture distributions, which is not equivalent to Euclidean distance, or any type of distance in general. Specifically, our proposed method deals with discrete count data with extra zero inflation, which makes it even more inappropriate to use distance as a measure of clustering performance.

In model-based clustering, there are two types of commonly used methods for determining K. One type of methods choose K based on information criterion such as Bayesian information criterion (BIC) (Fraley and Raftery, 1998). Such methods require performing model-based clustering at each

possible K, and even with a smaller range of possible K values, running the entire EM algorithm at each value is computationally intensive. In addition, such information-criterion-based methods often results in small K because the penalty is high due to the high-dimensionality of model parameters. Another type of methods use the idea of hybrid clustering, and start from a number of clusters K_0 which is much smaller than G while bigger than the potential values of K. Then, hybrid clustering methods merge two clusters at a time until all clusters are merged (Chen *et al.*, 2005). The hybrid clustering methods are more computationally efficient because they do not need to perform the whole algorithm separately for each $K = 1, \cdots, K_0$. Such methods have been commonly used in model-based clustering (J. van der Laan and Pollard, 2003; Chipman and Tibshirani, 2005; AlmodoÌvar-Rivera and Maitra, 2020).

As mentioned in Section 2.4 of the main text, we utilized a hybrid clustering algorithm to determine number of clusters. This method is based on the idea of sequential likelihood ratio tests. The details are listed below:

Algorithm 3: Hierarchical merging based on likelihood ratio test

1. First perform our EM or SA algorithm on K_0 clusters with K_0 large enough. We use $K_0 = \max(\lfloor\sqrt{G}\rfloor, 50)$ as indicated in (AlmodoÌvar-Rivera and Maitra, 2020);

2. After $(K_0 - K)$ merging steps where K clusters are left ($2 \leq K \leq K_0$), for each possible pair of clusters $(i, j), 1 \leq i < j \leq K$, find MLE and compute the total log likelihood $l_{ij} = l_i + l_j$ of this pair. Try merging the pair together, then find MLE of this new cluster and the corresponding log-likelihood l'_{ij};

3. Find the pair with the minimum loss ($\underset{i,j}{\text{argmin}}\{l_{ij} - l'_{ij}\}$), and merge the corresponding two clusters. This is the optimal merge from K to $(K-1)$ clusters. Record the p-value of the likelihood ratio test on this merging as $P_{(K-1)}$;

4. In the end we obtain a vector $\underset{\sim}{P} = (P_1, \cdots, P_{(K_0-1)})$. P_K close to 0 indicate rejections of null hypothesis, i.e. not merging from $(K+1)$ to K clusters. We take the smallest K with p-value $P_K > 0.01$ as the optimal number of clusters, which suggests that merging is no longer appropriate for all $k \in \{K, K-1, \cdots, 2\}$.

Note that this method is still time consuming, especially when starting with a relatively large $\max(\lfloor\sqrt{G}\rfloor, 50)$. Therefore we implemented this algorithm in our R package *PHclust*, with a parameter *Kstart* specifying the initial K_0. User can either choose to specify their own choice of K_0 by prior knowledge, or use $\max(\lfloor\sqrt{G}\rfloor, 50)$ by default.

5 Simulation Results for Unequal Cluster Size

In this section, we investigate the performance of clustering algorithms when the sizes of different clusters are quite different. One such situation is that all microbial features are included in cluster analysis while only a small proportion (say, 10%) are differentially abundant. Although the non-differentially abundant features may not be of interest, they are one big cluster with treatment effects being a vector of zeros.

In this simulation study, we set $p_1 = p_2 = 0.05$ and $p_3 = 0.9$. Here p_1, p_2 correspond to two clusters with different treatment effects, and p_3 corresponds to one cluster consisting of non-differential features whose abundance levels don't change across treatments.

We use the similar default simulation settings as in Section 3.1 of the main text. The only difference is that there are only 3 clusters (5%, 5%, 90%), and we set $\underset{\sim}{\delta}_k = (\delta_{k1}, \delta_{k2}, \delta_{k3})$ according to the following table.

Cluster k	1	2	3
δ_{k1}	1	-1	0
δ_{k2}	0	0	0
δ_{k3}	-1	1	0

Note that the third class with $\underset{\sim}{\delta}_3 = (0, 0, 0)$ consists of "noisy" features that are not differentially abundant across treatments. Similar to Section 3 in the main text, we apply the following 5 methods to compare their performance, each with 5 random starting points:

- Poisson hurdle model-based clustering with EM algorithm (PH-EM)
- Poisson hurdle model-based clustering with simulated annealing (PH-SA)
- Poisson model-based clustering (MB-Poisson)
- negative binomial model-based clustering (MB-NB)
- K-means clustering with Euclidean distance (K-means)

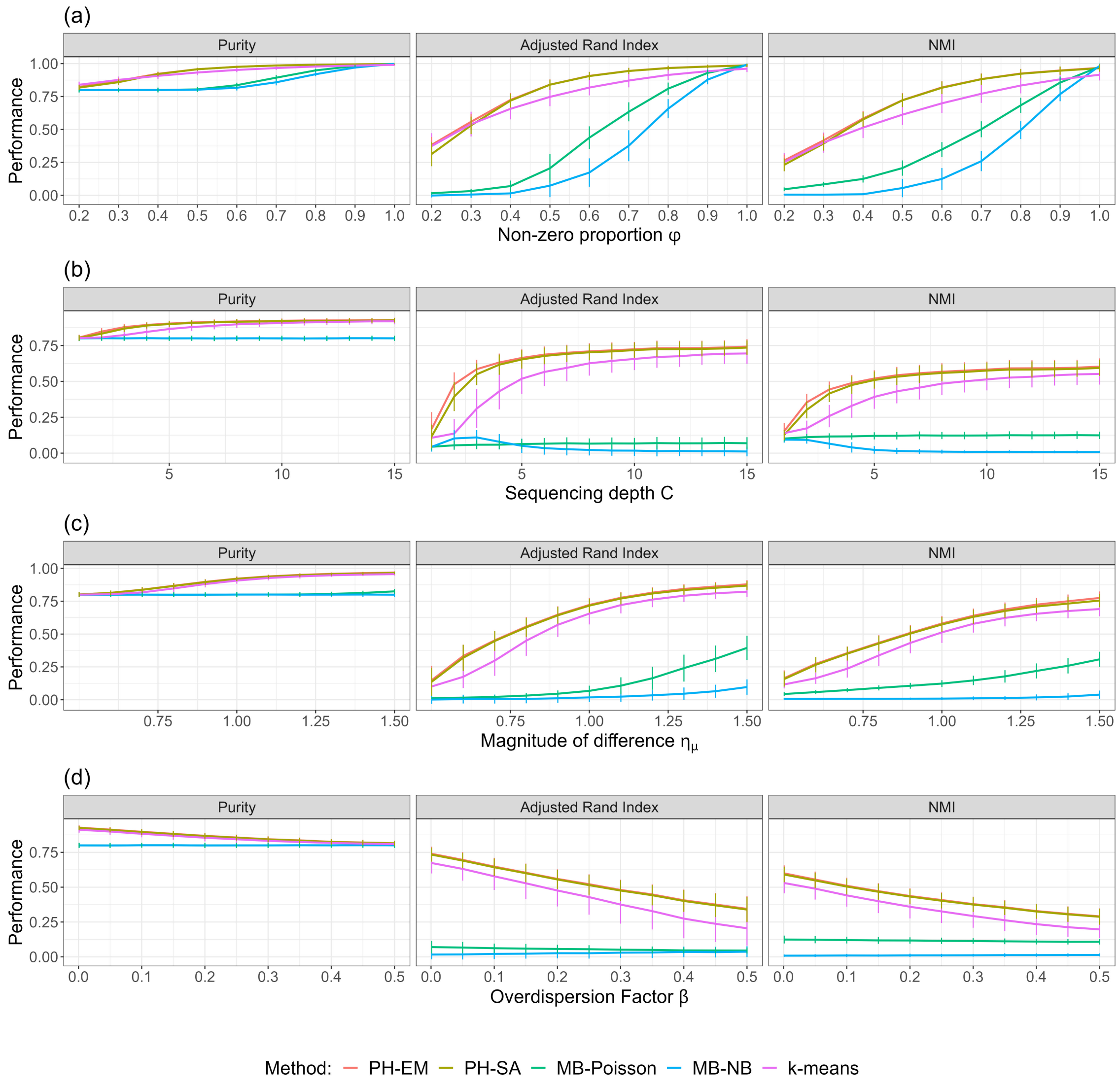

Clustering performance for simulation settings with unequal cluster sizes. For each parameter setting, 1000 datasets with dimension 1000×15 were simulated, and clustering performance are evaluated based on purity, adjusted Rand Index and NMI. The line charts plot the average scores across the 1000 datasets, while the vertical bars represent the standard error.
(The curves for $PH-EM$ and $PH-SA$ almost overlap.)
The default setting is $\phi = 0.4, C = 10, \eta_\mu = 1, \beta = 0.02$. Each plot varies one parameter at a time.
(a): Non zero proportion ϕ increases from 0.2 to 1
(b): Sequencing depth C increases from 1 to 15
(c): Magnitude of difference η_μ increases from 0.5 to 1.5
(d): Overdispersion factor β increases from 0 to 0.5

Note that the three true clusters are extremely unbalanced with one cluster containing 90% of features. Because of this, the *Purity*, which measures how "pure" each cluster is, obtains very large values compared to the other two metrics.

It is clear that the performance of our methods (PH-EM and PH-SA) still strongly dominates

the other competing methods. Also, comparing to the case of equal cluster size in Figure 1 of Section 3.2 in main text and Supplementary Figure 1, our methods still have similar, or even better, performance in this unequal cluster size scenario. The above findings validated the effectiveness of our method in both equal and unequal cluster sizes.

In practice, we recommend users not to include such high proportion of background noise but only use differentially abundant features for cluster analysis. Although including non-differentially abundant features has little influence on the performance of our clustering method, the computational cost is very high.

6 Evaluation Criteria

In our simulation studies (see Section 3.2 in the main text), the following three criteria were used to measure the concordance between different clustering results and the true cluster labels, with higher values indicating better performance:

- *Purity*:

  Purity measures how "pure" the clusters are, i.e. to what extent each cluster contains a single true class. Its formula is given by

$$Purity(\Omega, C) = \frac{1}{n} \sum_j \max_k |W_j \cap C_k|$$

  Where $\Omega = \{W_1, \cdots, W_J\}$ is the clustering results, $C = \{C_1, \cdots, C_K\}$ is the true cluster labels, and n is the total number of observations.

- *Adjusted Rand Index (ARI)*:

  The Rand Index was developed as a measure of similarity between two data clusterings. It calculates the proportion of pairs of features that are "correctly" assigned. Let $X = (x_1, \cdots, x_n)$ represent clustering results and $Y = (y_1, \cdots, y_n)$ represent true cluster labels. Define sets $A = \{(i,j), i < j : x_i = x_j \ \& \ y_i = y_j\}$ and $B = \{(i,j), i < j : x_i \neq x_j \ \& \ y_i \neq y_j\}$. Then,

$$Rand\ Index = \frac{|A| + |B|}{n(n-1)/2}.$$

  We use a slightly adjusted version by Hubert and Arabie (1985).

- *Normalized Mutual Information (NMI)*:

  Mutual information (MI) measures the shared information between two partitions. The normalized version of it (NMI) is usually preferred, which scaled the MI values to be within $[0, 1]$. The detailed formula for NMI can be found in Strehl and Ghosh (2002).

7 Convergence of the EM algorithm

In this section we investigate the computational time and the convergence of our EM algorithm. Based on a simulation setting of $G = 1000$ features, $I * J = 3 * 5 = 15$ samples, $I = 3$ treatments groups, and $K = 7$ real clusters, we plot the computational time of each single EM iteration (i.e., combination of 1 E-step and 1 M-step in Algorithm 1 of the main text) using 1000 simulated datasets in the following histogram. We used a hard threshold of 10 minimum iterations, so $> 10,000$ data points were used to make this histogram:

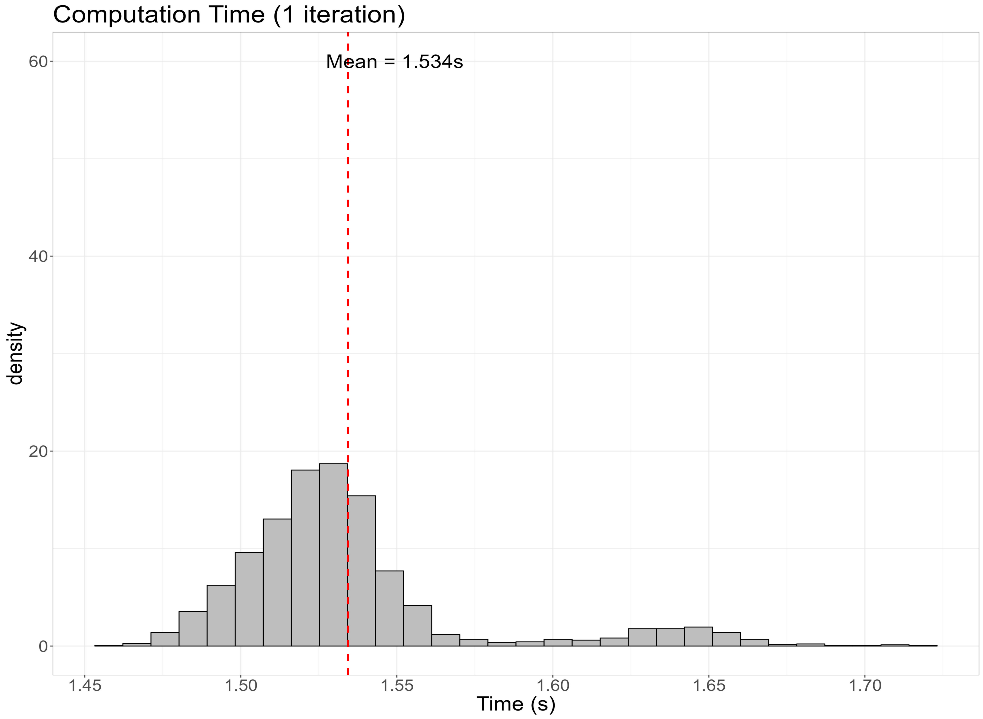

Computational time (s)

It took about 1.5 seconds on average for a single iteration for our simulation setting. The computational complexity for a single iteration is about $O(K(G+2I))$. The user may approximate estimation the running time accordingly for their datasets.

To investigate the convergence of the EM algorithm, we summarize the number of iterations that each full EM algorithm took across 1000 simulated datasets from the default setting in the table below: (Note that the the stopping rule is "log-likelihood change $< 0.0001\%$", along a minimum of 10 iterations).

# of Iterations	$10 \sim 20$	$20 \sim 30$	$30 \sim 40$	> 40
Proportions (%)	93.9	5.3	0.6	0.2

Table 1: The number of iterations until convergence of the EM algorithm

As shown on the table above, the EM algorithm converged within 20 iterations 93.9% of the time.

8 Extension of Model-based Negative Binomial Hurdle Model

In this section, we discuss the extension of our hurdle-model based clustering method to Negative Binomial hurdle models, which is mentioned in Section 5 of the main text. We also explain why we recommend the Poisson Hurdle model-based clustering for now.

We consider a Negative Binomial hurdle model, in which both the mean and overdispersion parameter depend on the feature abundance as follows, for modeling observation N_{gij} in cluster k, treatment i:

$$
\begin{aligned}
f_k(N_{gij}) &= \begin{cases} 1 - q_{kij}, N_{gij} = 0 \\ q_{kij} \times \dfrac{1}{1-(1-p)^r} \times \dfrac{\Gamma(r+N_{gij})}{\Gamma(N_{gij}+1)\Gamma(r)} p^{N_{gij}} (1-p)^r, N_{gij} > 0 \end{cases} \\
q_{kij} &= \frac{1}{1+\exp[-(\gamma_{0ki}+\gamma_{1ki}s_{ij})]} \\
r &= \frac{1}{\beta_{ki}} \exp(s_{ij}+\mu_{ki}) \\
p &= \frac{\beta_{ki}\exp(\alpha_{gk})}{1+\beta_{ki}\exp(\alpha_{gk})}
\end{aligned}
$$

The above model partly coincides with the fixed effect negative binomial regression model described in Allison and Waterman (2002). Under this model, the mean and variance parameters for the Negative Binomial part can be represented by:

$$
\begin{aligned}
E(N_{gij}) &= \exp(s_{ij} + \alpha_{gk} + \mu_{ki}) \\
Var(N_{gij}) &= [1 + \beta_{ki}\exp(\alpha_{gk})]E(N_{gij})
\end{aligned}
$$

With this negative binomial hurdle model, the mean is modeled the same as in our Poisson hurdle model, but there is an additional overdispersion parameter in each cluster k and treatment i that depends on the feature abundance α_{gk} and an unknown constant β_{ki}.

Since this model assumes a relationship between feature abundance α_{gk} and overdispersion, some commonly used methods that separately estimate dispersion may not be effective, for instance the pseudo-likelihood Lu *et al.* (2005) or quasi-likelihood (Nelder and Lee, 1992; Robinson and Smyth, 2007) method. Here we use a direct maximum likelihood approach to optimize over the parameters:

1. Because the number of features G is large compared to K and I, for the ease of computation we treat α_{gk} as the same ($\alpha_{gk} \equiv \alpha_g$) for all clusters k. We estimate α_g by the log upper quartile for each feature g at the very beginning, and then treat the estimates as known throughout the optimization. The usage of log upper quartile is to avoid the possible disturbance by too many zeros.

2. Similar to what we did in Section 2 of the Supplementary Materials, and since we estimated α's in advance, we can separately optimize the log likelihood in each combination of k and i. Let $l_{ki}(\beta_{ki}, \mu_{ki}) = l_{1ki}(\gamma_{0ki}, \gamma_{1ki}) + l_{2ki}(\beta_{ki}, \mu_{ki})$ denote the total log likelihood for cluster k, treatment i. We can optimize l_{1ki} following the exact same procedure as in Section 2 of the Supplementary Materials. The optimization for β_{ki} and μ_{ki} is much more tedious, but similar

to what we did before, we can use a coordinate descent algorithm to optimize over the two sets of parameters β_{ki}'s and μ_{ki}'s. :

$$
\begin{aligned}
l_{2ki}(\beta_{ki}, \mu_{ki}) = \sum_g Z_{gk} \sum_{j:N_{gij}\neq 0} \{ & -\log[1 - (1 + \beta_{ki} \exp(\alpha_g))^{-\frac{1}{\beta_{ki}} \exp(s_{ij}+\mu_{ki})}] + \\
& \log \Gamma(\frac{\exp(s_{ij} + \mu_{ki})}{\beta_{ki}} + N_{gij}) - \log \Gamma(\frac{\exp(s_{ij} + \mu_{ki})}{\beta_{ki}}) \\
& N_{gij} \log \frac{\beta_{ki} \exp(\alpha_g)}{1 + \beta_{ki} \exp(\alpha_g)} - \frac{\exp(s_{ij} + \mu_{ki})}{\beta_{ki}} \log(1 + \beta_{ki} \exp(\alpha_g))\} \\
\frac{\partial l_{2ki}}{\partial \beta_{ki}} = 0 & \\
\frac{\partial l_{2ki}}{\partial \mu_{ki}} = 0 &
\end{aligned}
$$

The problem again reduces to multiple univariate optimization problems, and can be solved numerically.

We compare the performance of Negative Binomial Hurdle models with the Poisson Hurdle models using the same simulation setting as in Section 3.1 of the main text, where $n_\mu = 1, C = 10, \phi = 0.4$, and overdispersion parameter β varying from 0 to 0.5 (overdispersion rate 0 to $0.5 * \exp(1.2) = 1.66$). Note that we use a common β instead of β_{ki} to simulate data, which is a special case for the model settings above.

We compare the clustering performance from the following 4 methods:

- Poisson hurdle model-based clustering with EM algorithm (PH-EM);
- Negative Binomial hurdle model-based clustering with EM algorithm (NBH-EM), the model we introduce in this section;
- Poisson model-based clustering (MB-Poisson);
- Negative Binomial model-based clustering (MB-NB)

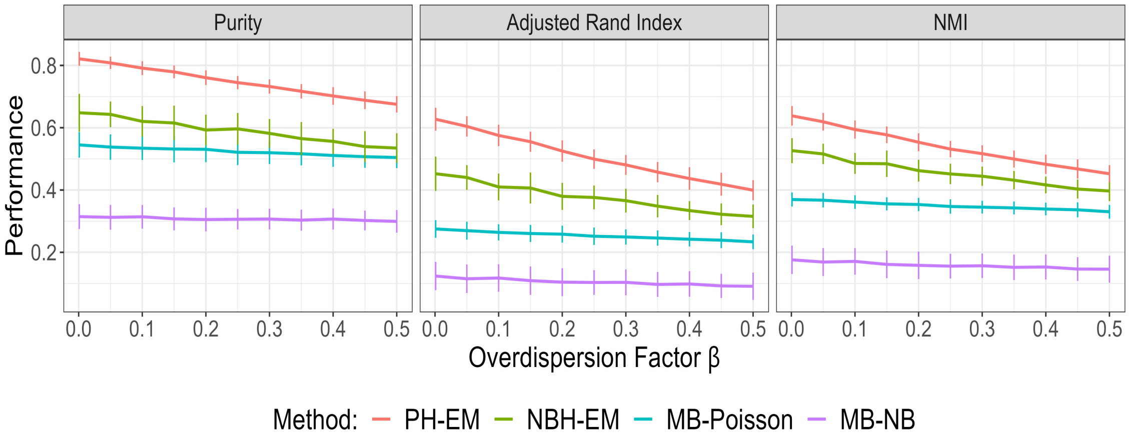

Comparison of clustering results using different model-based algorithms.
(Due to the high computational time of NBH model, we only did 100 simulations for each setting.)

As shown from the above figure, even when data were simulated from negative binomial hurdle distributions, the performance from PH-EM is still the best among all four methods. Moreover, the results from MB-Poisson are also better than results from MB-NB. We examined the parameter estimation and found that the estimation of overdispersion parameters is very unsteady and inaccurate, both in the NBH-EM model and in the MB-NB model from Si *et al.* (2014). Additionally, due to the complexity in optimizing negative binomial likelihood, the computation time for NBH-EM model is much longer ($\sim$ 100 times) than the Poisson hurdle model.

Considering both the unsatisfying performance and computational burden, we decided not to recommend the negative binomial hurdle model-based clustering in the main text.

9 Supplementary Figure 1

Clustering results for simulated datasets with $q_{kij} = \phi, \forall k, i$, using purity and adjusted Rand Index as the performance measure:

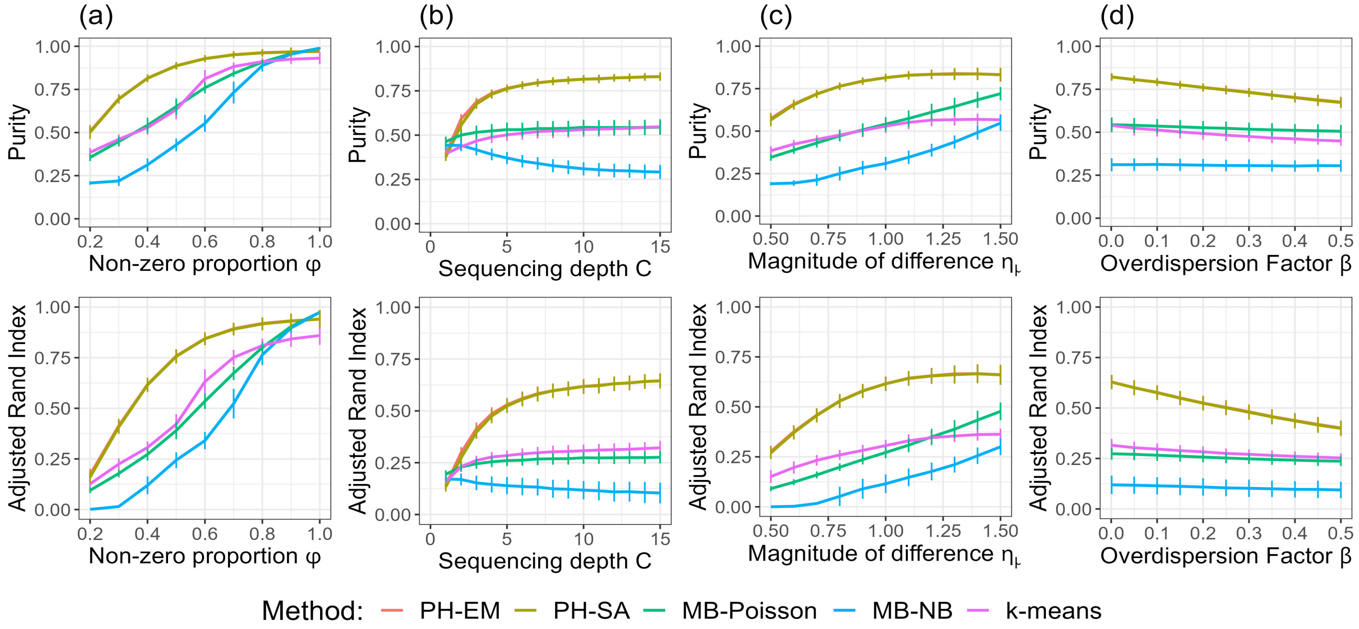

Figure 1: Simulation results for settings with constant zero inflation rate. For each parameter setting, 1000 datasets with dimension 1000×15 were simulated. The line charts plot the average scores (purity or adjusted Rand Index) across the 1000 datasets, while the vertical bars represent the standard error.
(The curves for $PH - EM$ and $PH - SA$ almost coincide)
The default setting is $\phi = 0.4, C = 10, \eta_\mu = 1, \beta = 0.02$. Each plot varies one parameter at a time.
(a): Non zero proportion ϕ increases from 0.2 to 1
(b): Sequencing depth C increases from 1 to 15
(c): Magnitude of difference η_μ increases from 0.5 to 1.5
(d): Overdispersion factor β increases from 0 to 0.5

10 Supplementary Figure 2

Clustering results for simulated datasets where zero-inflating structure significantly differs among clusters (see section 3.1 in main text for details):

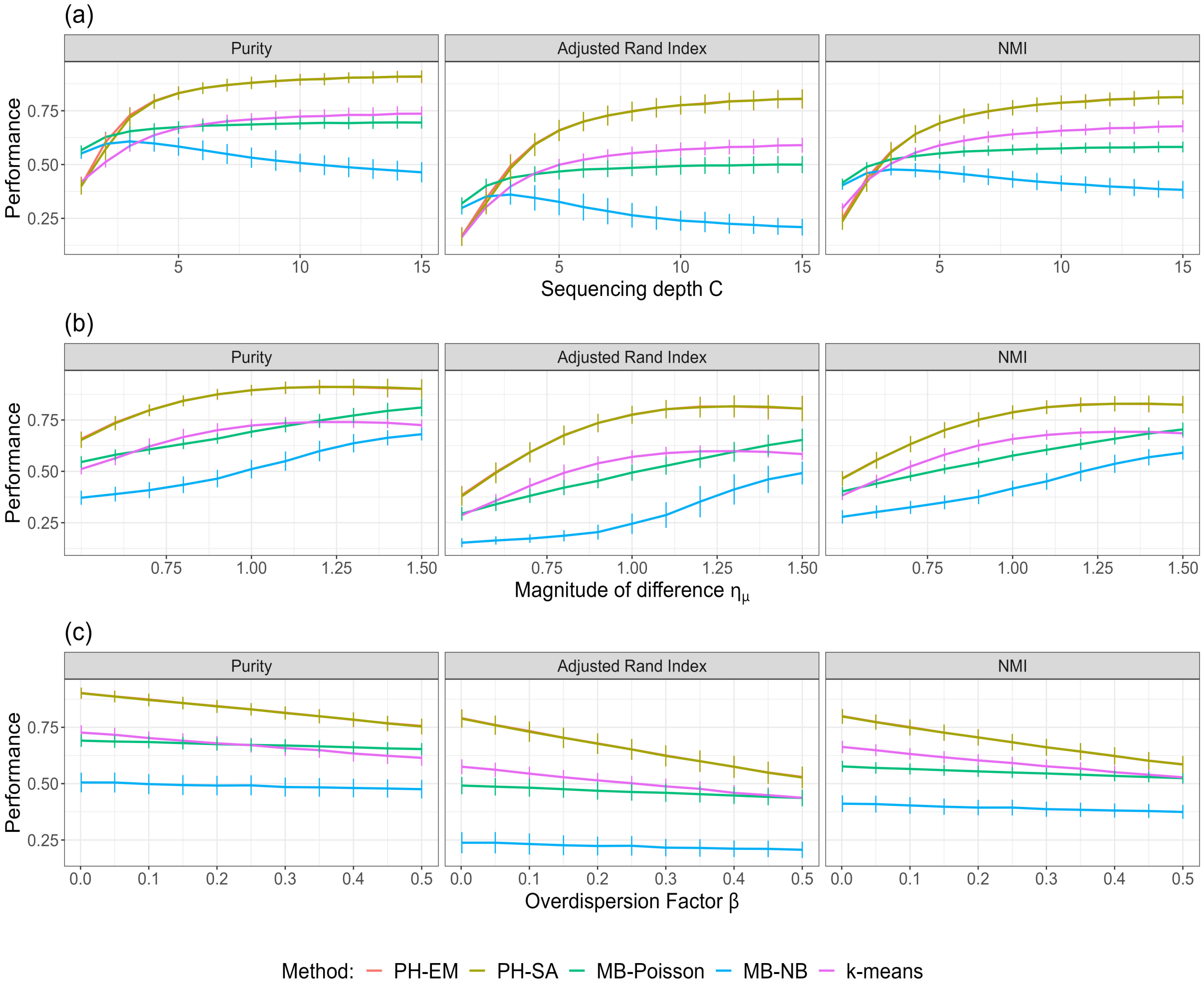

Figure 2: Simulation results for settings with varying zero inflation rates across clusters (and treatment). For each parameter setting, 1000 datasets with dimension 1000×15 were simulated, and clustering performance are evaluated based on purity, adjusted Rand Index and NMI. The line charts plot the average scores across the 1000 datasets, while the vertical bars represent the standard error.
(The curves for $PH - EM$ and $PH - SA$ almost coincide)
The default setting is $\phi = 0.4, C = 10, \eta_\mu = 1, \beta = 0.02$. Each plot varies one parameter at a time.
(a): Sequencing depth C increases from 1 to 15
(b): Magnitude of difference η_μ increases from 0.5 to 1.5
(c): Overdispersion factor β increases from 0 to 0.5

11 Supplementary Figure 3

Evaluation of initialization methods, using purity and adjusted Rand Index as the performance measure:

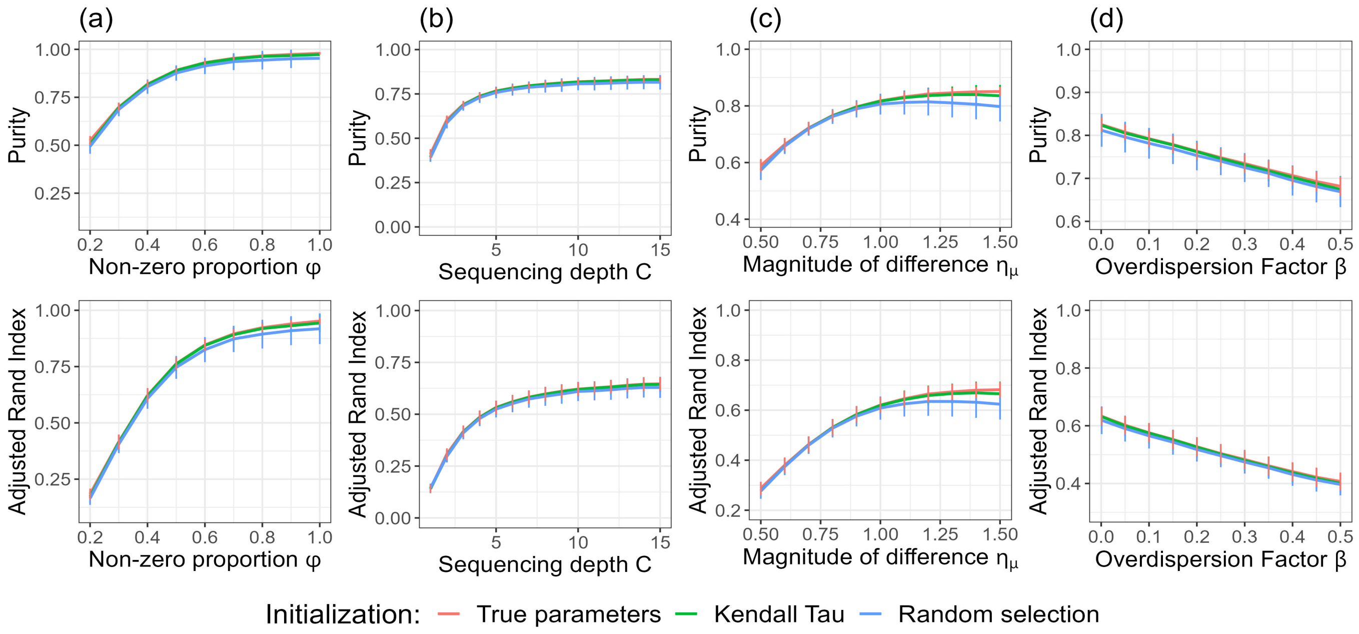

Figure 3: Comparison of initialization method using true parameters, Algorithm 2 (Kendall Tau), and completely random selection. Simulation results are based on different sets of parameters and $\bar{q}_{ki} = \phi$. For each parameter setting, 1000 datasets with dimension 1000×15 were simulated. The line chart plots the average score across the 1000 datasets, while the vertical bar represents the standard error.
(See Supplementary Figure 1 for parameter settings)

12 Supplementary Figure 4

Evaluation of the multiple starting methodology with different number of starts:

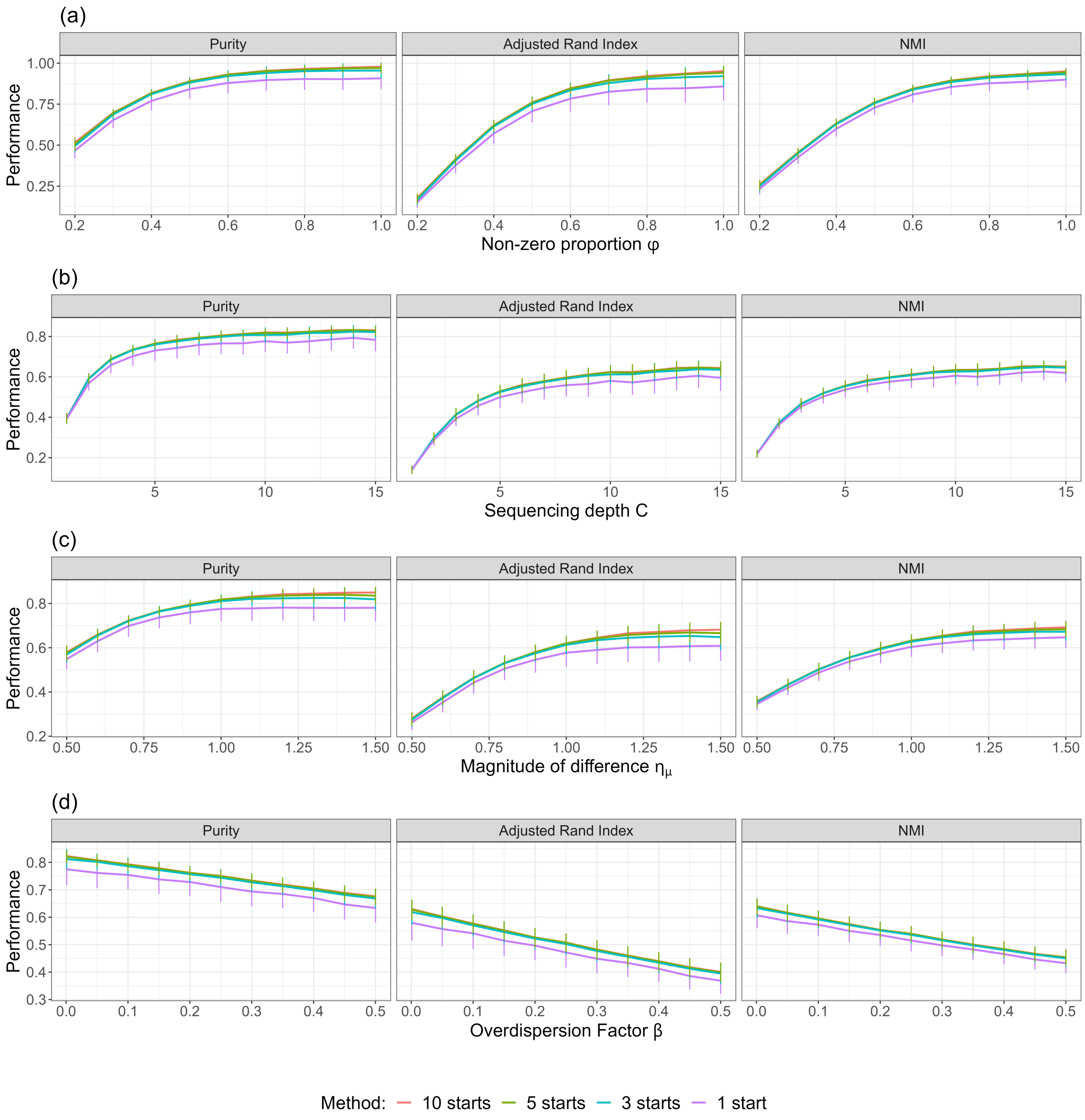

Figure 4: Evaluation on different number of starts. Simulation results are based on settings with constant zero-inflation rate. For each parameter setting, 1000 datasets with dimension 1000×15 were simulated, and clustering performance are evaluated based on purity, adjusted Rand Index and NMI. The line charts plot the average scores across the 1000 datasets, while the vertical bars represent the standard error.
(See Supplementary Figure 1 for parameter settings)

13 Supplementary Figure 5

The mean feature abundance profile for clustering results of PH-SA and k-means:

(a)

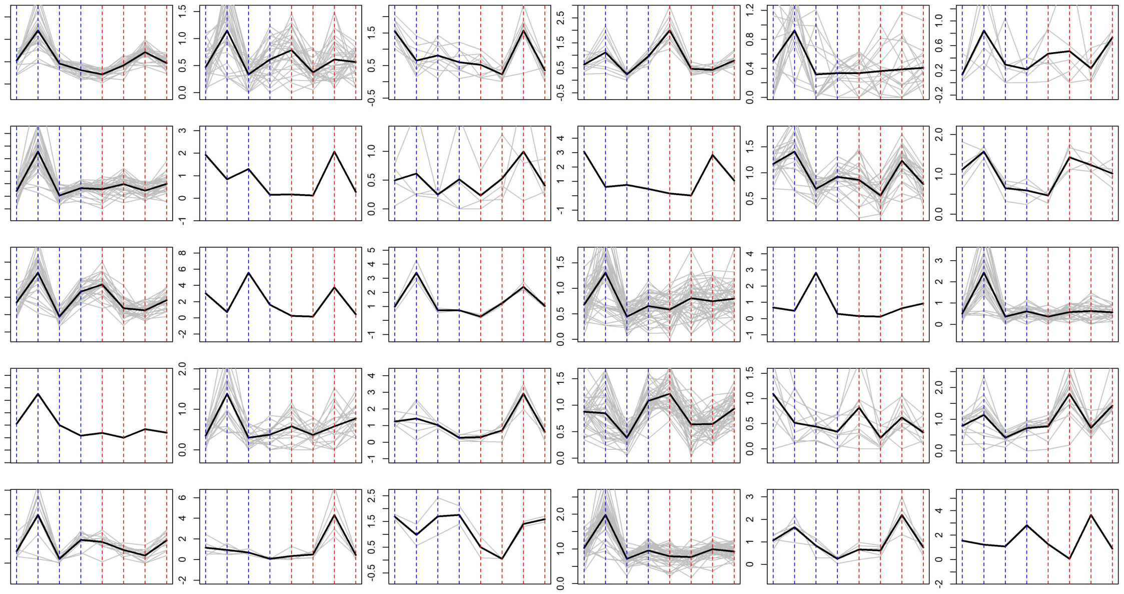

(b)

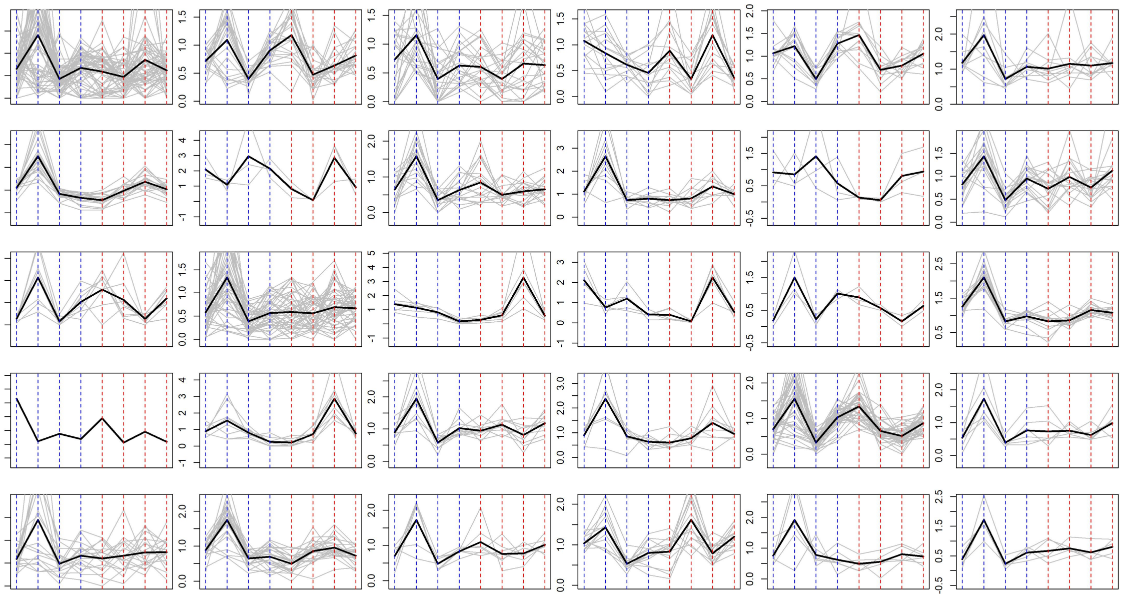

Figure 5: ASV abundance profile for: (a) Poisson hurdle clustering with simulated annealing (PH-SA) and (b) K-means clustering. Each subplot correspond to a cluster, x-axis corresponds to the 8 treatment groups (2 nitrogen levels with 4 chronically ordered dates, 1∼4 correspond to high nitrogen, 5∼8 correspond to low nitrogen) and y-axis represents the abundance level. Each grey line corresponds to a moment-based estimate of feature abundance level for an ASV, and black line represents the geometric mean in each cluster.

14 Supplementary Figure 6

The abundance of nitrogen-fixing gene:

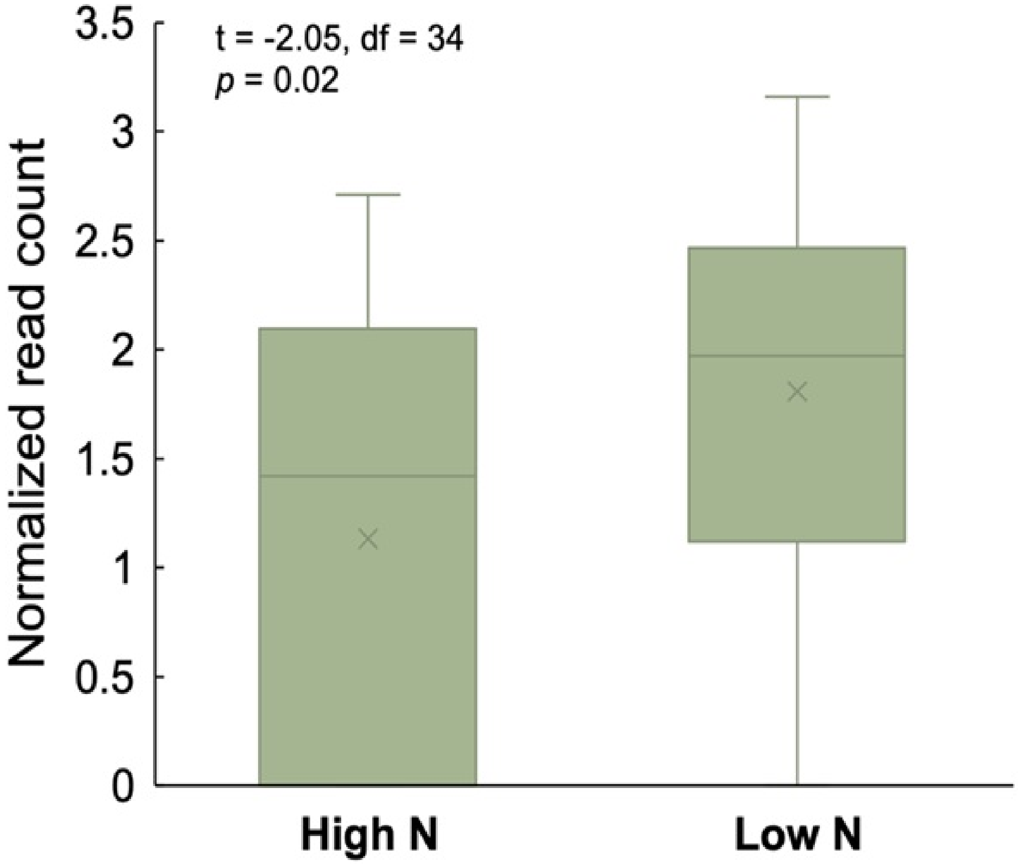

Figure 6: Nitrogen-fixing (*nifHDK*) gene abundance in the sorghum rhizosphere from both 2016 and 2017 metagenomes. Read abundances were Deseq2-normalized and log2 transformed and compared with a t-test.

References

Allison, P. D. and Waterman, R. P. (2002). Fixed–effects negative binomial regression models. *Sociological Methodology*, **32**(1), 247–265.

AlmodoÌvar-Rivera, I. A. and Maitra, R. (2020). Kernel-estimated nonparametric overlap-based syncytial clustering. *Journal of Machine Learning Research*, **21**(122), 1–54.

Chen, B., Tai, P. C., Harrison, R., and Yi Pan (2005). Novel hybrid hierarchical-k-means clustering method (h-k-means) for microarray analysis. In *2005 IEEE Computational Systems Bioinformatics Conference - Workshops (CSBW'05)*, pages 105–108.

Chipman, H. and Tibshirani, R. (2005). Hybrid hierarchical clustering with applications to microarray data. *Biostatistics*, **7**(2), 286–301.

Croux, C. and Dehon, C. (2010). Influence functions of the spearman and kendall correlation measures. *Statistical Methods & Applications*, **19**(4), 497–515.

Fraley, C. and Raftery, A. E. (1998). How Many Clusters? Which Clustering Method? Answers Via Model-Based Cluster Analysis. *The Computer Journal*, **41**(8), 578–588.

Hubert, L. and Arabie, P. (1985). Comparing partitions. *Journal of Classification*, **2**(1), 193–218.

J. van der Laan, M. and Pollard, K. S. (2003). A new algorithm for hybrid hierarchical clustering with visualization and the bootstrap. *Journal of Statistical Planning and Inference*, **117**(2), 275 – 303.

Lu, J., Tomfohr, J. K., and Kepler, T. B. (2005). Identifying differential expression in multiple sage libraries: an overdispersed log-linear model approach. *BMC Bioinformatics*, **6**(1), 165.

Nelder, J. A. and Lee, Y. (1992). Likelihood, quasi-likelihood and pseudolikelihood: Some comparisons. *Journal of the Royal Statistical Society: Series B (Methodological)*, **54**(1), 273–284.

Robinson, M. D. and Smyth, G. K. (2007). Small-sample estimation of negative binomial dispersion, with applications to SAGE data. *Biostatistics*, **9**(2), 321–332.

Rousseeuw, P. J. (1987). Silhouettes: A graphical aid to the interpretation and validation of cluster analysis. *Journal of Computational and Applied Mathematics*, **20**, 53–65.

Si, Y., Liu, P., Li, P., and Brutnell, T. P. (2014). Model-based clustering for RNA-seq data. *Bioinformatics*, **30**(2), 197–205.

Strehl, A. and Ghosh, J. (2002). Cluster ensembles - a knowledge reuse framework for combining multiple partitions. *Journal of Machine Learning Research*, **3**, 583–617.

Tibshirani, R., Walther, G., and Hastie, T. (2001). Estimating the number of clusters in a data set via the gap statistic. *Journal of the Royal Statistical Society: Series B (Statistical Methodology)*, **63**(2), 411–423.
