# Supplementary figures and images for: Poisson hurdle model-based method for clustering microbiome features
Source: Bioinformatics. 2022 Dec 5;39(1):btac782. doi: 10.1093/bioinformatics/btac782 (PMC9825753; doi:10.1093/bioinformatics/btac782)

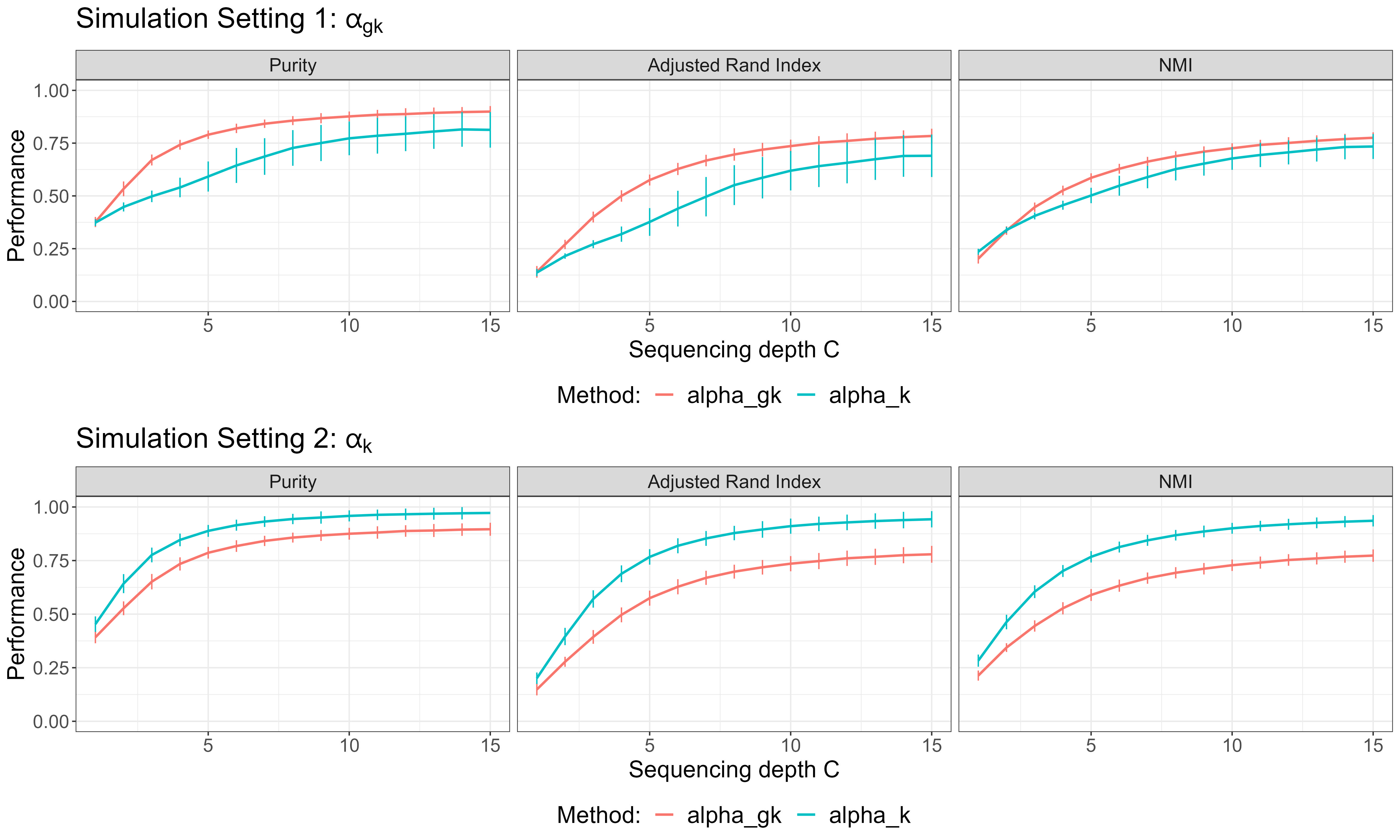

Supplement: btac782_Supplementary_Data [file btac782_supplementary_data.zip › gk_k.jpg]
